# BASIC PENTACYSTEINE1 regulates *ABI4* by modification of two histone marks H3K27me3 and H3ac during early seed development of *Medicago truncatula*

**DOI:** 10.3389/fpls.2024.1395379

**Published:** 2024-06-10

**Authors:** Thi Thu Dang, David Lalanne, Joseph Ly Vu, Benoit Ly Vu, Johan Defaye, Jerome Verdier, Olivier Leprince, Julia Buitink

**Affiliations:** ^1^ INRAE, Institut Agro, Univ Angers, Institut de Recherche en Horticulture et Semences, SFR QUASAV, Angers, France; ^2^ LIPME - Laboratoire des interactions plantes-microbes-environnement. UMR CNRS–INRAE, Castanet Tolosan, France

**Keywords:** BPC1, H3K27me3, H3Ac, longevity, seed development, *ABI4*

## Abstract

**Introduction:**

The production of highly vigorous seeds with high longevity is an important lever to increase crop production efficiency, but its acquisition during seed maturation is strongly influenced by the growth environment.

**Methods:**

An association rule learning approach discovered MtABI4, a known longevity regulator, as a gene with transcript levels associated with the environmentally-induced change in longevity. To understand the environmental sensitivity of *MtABI4* transcription, Yeast One-Hybrid identified a class I BASIC PENTACYSTEINE (MtBPC1) transcription factor as a putative upstream regulator. Its role in the regulation of *MtABI4* was further characterized.

**Results and discussion:**

Overexpression of MtBPC1 led to a modulation of *MtABI4* transcripts and its downstream targets. We show that MtBPC1 represses *MtABI4* transcription at the early stage of seed development through binding in the CT-rich motif in its promoter region. To achieve this, MtBPC1 interacts with SWINGER, a sub-unit of the PRC2 complex, and Sin3-associated peptide 18, a sub-unit of the Sin3-like deacetylation complex. Consistent with this, developmental and heat stress-induced changes in *MtABI4* transcript levels correlated with H3K27me3 and H3ac enrichment in the *MtABI4* promoter. Our finding reveals the importance of the combination of histone methylation and histone de-acetylation to silence *MtABI4* at the early stage of seed development and during heat stress.

## Introduction

High seed vigor is essential for the propagation and preservation of our genetic resources (Colville and Pritchard, 2019). The different traits that encompass seed vigor include the capacity and speed of germination, absence of dormancy, and the ability to remain alive in the dry state, referred to as longevity. The development of seeds is a process separated into two phases: embryogenesis and maturation, the latter being further divided into seed filling and late seed maturation. In *Medicago truncatula*, the transition from embryogenesis to seed filling occurs around 10–12 days after pollination (DAP), when cells stop dividing and start filling with storage reserves (Wang et al., 2012). During seed filling, seeds acquire their capacity to germinate and to tolerate drying, referred to as desiccation tolerance (DT). At the later stage of maturation, longevity is progressively acquired, after which the seed undergoes final maturation drying when the pods disconnect from the mother plant (Verdier et al., 2013; Righetti et al., 2015; Zinsmeister et al., 2016). In legumes, the environment during seed development has an important impact on the developmental program, not only leading to the premature arrest of the maturation program, but also affecting seed longevity (Kochanek et al., 2011; Delmas et al., 2013; Righetti et al., 2015; Zinsmeister et al., 2020; Chen et al., 2021). At the molecular level, the environmental factors impacting key signaling pathways in seed development include ROS production (Groot et al., 2012), phytohormone regulation (Clerkx et al., 2003; Liu et al., 2013), and plant DNA damage response (Waterworth et al., 2022). However, little is known about the genes that control seed vigor and how their regulation is perturbed by the environment. Yet, this understanding is crucial to improve the stability of seed traits in light of climate change.

Seed development is controlled by a network of transcription factors regulating downstream target genes. Some of the master regulators are part of the LAFL network, consisting of four transcription factors; three B3 domain factors (LEAFY COTYLEDON2 [LEC2], FUSCA3 [FUS3], ABSCISIC ACID INSENSITIVE3 [ABI3]) and LEC1 (a HEME ACTIVATED PROTEIN3 subunit of CCAAT-binding factors) (Giraudat et al., 1992; Lotan et al., 1998; Luerßen et al., 1998; Stone et al., 2001). In legumes, two other ABI transcription factors, ABSCISIC ACID INSENSITIVE4 (ABI4) and ABSCISIC ACID INSENSITIVE5 (ABI5), are additional important pleiotropic regulators. Defective mutants of both genes are severely affected in seed maturation, leading to decreased seed vigor, longevity, dormancy, and seed degreening (Zinsmeister et al., 2016, 2023). Besides the regulation of seed maturation, these transcription factors are also known to play a role in other processes. ABI5 is an important regulator of dormancy and post-germination in relation to abiotic stress, inducing radicle growth arrest upon water stress (Lopez-Molina et al., 2001; Brocard et al., 2002; Skubacz et al., 2016). The AP2/ERF domain transcription factor ABI4 was originally identified as one of the components of ABA signaling regulating seed germination (Finkelstein et al., 1998). However, ABI4 is involved in a wider range of processes including flowering control, root development, plant defense, antagonism between abscisic acid and gibberellins or lipid mobilization during seedling establishment (Penfield et al., 2006; Cantoro et al., 2013; León et al., 2013; Shu et al., 2016; Barczak-Brzyżek et al., 2017; Shu et al., 2018; Zhu et al., 2020). Recently, we identified a role for ABI4 in the regulation of dormancy and longevity, in part via the coordinated dismantlement of chloroplasts during seed development, to avoid damage and interference with the acquisition of seed vigor traits (Zinsmeister et al., 2023).

The vital and versatile functions that implicate ABI4 implies strict regulation (Chandrasekaran et al., 2020). Many upstream activators or suppressors have been identified during seed germination and early seedling development. WRKY8 is found to interact directly with *ABI4* through the W-box located in its promoter to stimulate *ABI4* expression. MYB96, an ABA-responsive R2R3-MYB type transcription factor, binds directly to the promoter of *ABI4* and enhances transcription (Lee et al., 2015). The MYB96-ABI4 module inhibits lipid mobilization in the embryo, thereby delaying seed germination under sub-optimal condition (Lee et al., 2015). In *Arabidopsis*, the RAV1 (RELATED TO ABI3/VP1) transcription factor interacts directly with the promoter region of *ABI4* and represses its transcription level (Feng et al., 2014). Recent studies highlight the importance of epigenetic regulation in controlling *ABI4* transcription. In *Arabidopsis*, the NODULIN HOMEOBOX gene (*AtNDX*), which is required for heterochromatin homeostasis binds to the 3`UTR of *AtABI4* and represses its transcription via the direct interaction with AtRING1A and AtRINGB, two key ubiquitinase enzymes of the PRC1 complex (Zhu et al., 2020).

The Polycomb repressive complex 2 (PRC2) has been well documented to be able to induce histone trimethylation of Lysine 27 (H3K27me3), resulting in chromatin modification and long-lasting gene repression (Mozgova et al., 2015). Several studies in *Arabidopsis* indicate that PRC2 recruits the GAGA binding motif protein BASIC PENTACYSTEINE (BPC) to the promoter of downstream targets (Hecker et al., 2015; Xiao et al., 2017; Li et al., 2019). BPCs contain conserved DNA binding domain with five conserved cysteine residues at the C-terminus (Meister et al., 2004) and play important roles in variety of developmental processes in plants, including the regulation of the expression of the seed developmental master regulators *LEC2*, *FUS3* and *ABI3* (Berger et al., 2011; Simonini et al., 2012; Simonini and Kater, 2014; Mu et al., 2017b; Wu et al., 2020). In roots, AtBPC4 binds directly at the CT-rich motif within the *ABI4* promoter and represses its transcription by recruiting SWINGER (SWN), a sub-unit of the PRC2 complex to modify the H3K27me3 state of *ABI4* (Mu et al., 2017b).

In *Drosophila*, the GAGA factor interacts with Sin3A Associated Protein 18 (SAP18), a subunit of the histone regulator Sin3-deacetylation complex to regulate the expression of homeotic genes (Espinás et al., 2000). The Sin3 complex comprises eight components conserved from yeast to human, including SIN3, HDAC1, HDAC2, RbAp46, RbAp48, SAP30, and SAP18, which is named from its small size, being 18 amino acids (Silverstein and Ekwall, 2005). Sin3-deacetylation complex removes the acetyl groups from histones, resulting in low accessibility of transcription factors and, therefore, reduced gene expression (De Ruijter et al., 2003).

In this study, to identify genes that could explain the effect of environment on seed maturation, we used the transcriptome dataset published in Righetti et al. (2015) to construct a directed multilevel network that highlights seed-enriched transcripts whose relative abundance is linked to environmental stress and/or longevity. *MtABI4* was identified as a gene that with decreased transcript levels that were associated with decreased longevity upon heat and water stress. Upstream regulators underlying the transcriptional modulation of *ABI4* upon environmental changes during seed development were further investigated. Using a Yeast One-Hybrid (Y1H) assay, a GAGA transcription factor, named MtBPC1, was identified to directly bind *MtABI4* promoter at the CT-rich motif to repress transcription. MtBPC1 was found to interact with MtSWN, a key component of the PRC2 complex, and MtSAP18, a sub-unit of the Sin3-deacetylation complex. Real-time quantitative PCR (RT-qPCR) and Chromatin immunoprecipitation-quantitative PCR (ChIP-qPCR) analyses demonstrated that there was a strong connection between *ABI4* gene expression and its H3K27me3 (trimethylation of histone H3 at lysine 27) and H3ac (histone H3 acetylation) promoter enrichment. Our finding reveals the importance of the combination of two epigenetic modifications, histone methylation, and histone de-acetylation, for efficient repression of *ABI4* expression at the early stage of seed development.

## Materials and methods

### Plasmid construction and plant material

To generate the over-expression lines of MtBPC1 in *Medicago truncatula*, the full coding sequence of *MtBPC1* was cloned into gateway vector pGWB6, driven by the 35S promoter. The stable transgenic R108 plants were generated by *Agrobacterium tumefaciens*-medicated transformation (strain AGL6), according to the protocol of Trinh et al. (1998). *M. truncatula* lines were cultivated in climatic chambers at standard (20/18°C) or heat stress (26/23°C) conditions according to the protocol described by Righetti et al. (2015). Flowers were tagged at pollination and harvested at different days after pollination. The genomic sequence of *MtBPC1* (MtrunA17_Chr8g0375531) was sent to the Samuel Robert Foundation (Oklahoma, 168 USA) for reverse screening to identify *Tnt1* insertion mutants. Three lines were obtained: NF3308 (*bpc1–1*), NF9344 (*bpc1–2*), and NF13785 (*bpc1–3*). *Tnt1* insertions and homozygous lines of the different mutants were verified by PCR with primers shown in **Supplementary Table S1**.

### RulNet analysis

The transcriptomes of *M. truncatula* seed development under different environmental growth conditions (standard, heat, cold, water stress) from Righetti et al. (2015) were used to extract gene transcripts that are preferentially expressed in seeds compared to other plant tissues (>5x, 740). The quantitative measures of longevity (P50) for the four environmental conditions were used together with the gene transcripts to infer a directed multilevel network of transcripts and seed longevity that were significantly impacted by heat, cold and water stress. The association rule learning algorithm describing rule semantics between attributes was used using the platform RulNet, dedicated to the inference and analysis of regulatory networks from qualitative and quantitative –omics data by means of rule discovery and statistical techniques (http://rulnet.isima.fr; Vincent et al., 2015). Separation in different maturation stages allowed for the identification of genes that differ only at a specific developmental stage (**Supplementary Figure 1A**). Only rules involving at least one of the central attributes are discovered. Data were first scaled and centered, and semantics were written allowing the discovery of links (rules) between variables according to their relative abundance among four different developmental stages (**Supplementary Figure S1B**). Support, confidence, and lift thresholds were set at 0.2, 0.8, and 1.5, respectively. Validated rules were used to visualize networks using CYTOSCAPE software v3.3.0 (Smoot et al., 2011). In the resulting network, red and blue edges indicate links discovered using the upregulated or down-regulated values, respectively.

### Alignment and phylogeny analysis

Five predicted *Medicago* BPCs protein sequences were aligned with seven identified BPCs *Arabidopsis* protein sequences used to generate the phylogenetic tree, using default settings (https://www.phylogeny.fr). The alignment of AtBPC1, AtBPC4, AtBPC6 and MtBPC1 was performed using https://tcoffee.crg.eu.

### Yeast one-hybrid

Yeast One-Hybrid library screening was performed following the protocol of Matchmaker Gold Yeast One-Hybrid Library Screening system (www.clontech.com). A 1300 bp fragment of the *MtABI4* promoter was cloned into the pBait-AbAi vector and transformed into Y1H Gold yeast as the reporter strain. To construct the library, cDNA was synthesized from a collection of seeds harvested at 20, 24 and 36 days after pollination (DAP) and mature seeds, purified and transformed into the pGADT7-Rec AD cloning vector. The screening process was carried out on a medium supplemented with the appropriate antibiotic according to the manufacturer’s protocol. Surviving colonies were re-cultured for plasmid purification. The cDNA of the positive clones was amplified by PCR with primers specific for pGADT7 vector (provided in the Kit). The PCR product was purified and sent for sequencing using the T7 primer. For single transformation, 60 bp of the *MtABI4* promoter sequence (**Supplementary Table S1**) was cloned into the pBait-AbAi vector as bait construct, and the coding sequence of *MtBPC1* was cloned into pGADT7-Rec AD cloning vector as prey construct. The transformation and selection were carried out similarly to library screening.

### Gene expression analysis

Total RNA was extracted from transformed or wildtype *M. truncatula* seeds collected at different stages of development under standard growth conditions (20°C) and heat stress (26°C) conditions using the NucleoSpin RNA Plus kit (Macherey Nagel) according to the manufacturer’s instructions. cDNA was synthesized using the Reverse Transcription system (iScriptTM cDNA synthesis kit, Bio-Rad). Quantitative Real-time PCR was performed using Sybr Green Master Mix (SYBR Green master mix, Bio-Rad) on a CFX96 real-time detection system (Bio-Rad Laboratories). *Actin11 (MtAct11)* gene (MtrunA17_Chr2g0278591) and *MtTCTP* gene (MtrunA17_Chr2g1006185) were used as internal controls (Zinsmeister et al., 2016). Primers used for Real-time PCR are listed in **Supplementary Table S1**. All data were analyzed using three biological replicates and two technical replicates retrieved from seed lots originating from 3–6 plants/genotype.

### DNA pull-down assay

A 60-bp fragment of the *MtABI4* promoter containing the CT-rich motif for the BPC1 binding site and a mutated version of this 60-bp fragment (**Supplementary Table S1**) were labeled with Biotin at the 5`end (www.eurofinsgenomics.eu). To obtain 4µg of double stranded 60-bp fragment, the sense and anti-sense strands were mixed with equal volume of 8 µg and incubated at 100°C for 1h. The coding sequence of MtBPC1 was cloned into the pGWB6 vector by LR reaction. *A. tumefaciens* transformed with the constructs was cultured overnight, and re-cultured on the next day until the OD600 reached 1 before infiltration. Empty pGWB6 and BPC1-pGWB6 vectors were infiltrated into 3-week-old *Nicotiana benthamiana* leaves by *A. tumefaciencs* transformation. *A. tumefaciens* transformed with P19, (pCB301 vector), which acts as a silencing inhibitor, was also cultured at OD600 = 1 and co-infiltrated with the empty pGWB6 or MtBPC1-pGWB6. Infected leaves were harvested 48 h after infiltration for protein extraction. Two leaf disks were ground into powder and incubated in 1 ml of PBS protein extraction buffer (10 mM NaH2PO4/Na2HPO4, pH 7.5, 140 mM NaCl) and protein cocktail inhibitor (complete EDTA free Protease Inhibitor Cocktail, Roche), (1 tablet/50 ml of 241 extraction buffer). Samples were kept on ice for 20 min and centrifugated at 11000g for 15 min, after which supernatants were collected. A reaction containing 40 µl of Streptavidin beads, 4 µg of double-stranded oligos (original or mutated), and 400 µg of protein extract in 500 µl of PBS buffer went under gentle rotation at 4°C. After 2h, the streptavidin beads were washed three times with PBS buffer and denatured to extract bound proteins that were processed using Western Blot analysis.

### Transient expression and β-Glucuronidase (GUS) staining

A 1300-bp fragment of *MtABI4* promoter was constructed into pKGWFS7 carrying the *GUS* gene (*pMtABI4::GUS*) and the coding sequence of *BPC1* was constructed into the pGWB6 vector (CaMV35S::MtBPC1) by Gateway LR reaction. The *MtABI4* promoter-pKGWFS7, empty pGWB6, and BPC1-pGWB6 plasmids were transformed into *A. tumefaciens* that was thereafter grown overnight. The culture was adjusted to an OD600 of 0.5 before infiltration into *N. Benthamiana* leaves. The co-infiltration was performed with *MtABI4* promoter-*GUS* + empty pGWB6 (Green fluorescent protein (GFP)) or *MtABI4* promoter-*GUS* + MtBPC1-GFP in pGWB6, together with P19 as a silencing inhibitor. To examine the transient expression of *MtABI4* by MtBPC1, transcript levels of *GUS* were measured in 24h-infiltrated leaves using qRT-PCR. The relative expression of *GUS* was normalized to *N. Benthamiana* house-keeping genes, *EF1* and *L25*.

For GUS staining, *N. benthamiana* leaves were harvested 36–48 h after infiltration, placed on a Petri dish containing 90% acetone, and stored on ice until all the samples were collected. The samples were incubated at room temperature for 20 min. The staining buffer was freshly prepared (50 mM phosphate buffer, 2 mM ferricyanide potassium, and 0.2% Triton X100) and kept on ice. After incubation, acetone was replaced with 1 ml of iced staining buffer. After 5 min, the staining buffer was replaced with 1 ml of staining solution (200 µl of 5-bromo-4-chloro-3-indolyl-β-D-glucuronic acid cyclohexylammonium salt (X-Gluc) solution in 4.8 ml of staining buffer), followed by 15 min cold vacuum infiltration. Samples were incubated overnight at 37°C. The next day, the leaf tissues were washed several times with 70% ethanol at room temperature until they became colorless.

### Yeast two-hybrid

Coding sequences of *MtCLF* (*CURLY LEAF*, MtrunA17_Chr5g0401921), *MtFIE* (*FERTILIZATION-INDEPENDENT ENDOSPERM*, MtrunA17_Chr1g10587912), *MtVRN2* (*REDUCED VERNALIZATION RESPONSE 2*, MtrunA17_Ch5g0399611), *MtMSI1* (*MULTICOPY SUPRESSOR OF IRA1*, MtrunA17_Chr4g0052291) *MtSWN* (MtrunA17_Chr1g0194631), *MtSAP18 (*MtrunA17_Chr8g0351071), *MtLHP1* (*LIKE HETEROCHROMATIN PROTEIN 1*, MtrunA17_Chr8g033787) and *MtBPC1* (MtrunA17_Chr8g0375531) were cloned into the pENTR vector (Invitrogen, CA, USA) and the entry clones were confirmed by DNA sequencing. The destination bait vector BTM116-GW and prey vector Pvp16-GW were generated by the Gateway LR reaction according to the manufacturer’s protocol. The primer sequences of each gene are listed in **Supplementary Table S1**. The transformation and selection processes were performed according to Zhu et al. (2010). The inhibitor of the HIS3 reporter gene, 3-Amino-1,2,4 (3AT), was added with a concentration of 2 mM.

### Bimolecular fluorescence complementation assay

The entry vector carrying the CDS sequence of *MtBPC1* was cloned into the pDEST-VYNE(R)GW vector by Gateway LR reaction according to the manufacturers’ instructions (*Invitrogen*). Similarly, entry vectors containing the coding sequences of *MtSWN*, *MtSAP18* and *MtVRN2* were ligated into pDEST-VYCEGW vector (Gehl et al., 2009). The constructs were co-infiltrated into *N. Benthamiana* leaves according to the above protocol. 24h after infiltration, infected leaves were cut and observed under the epi-fluorescence microscope Zeiss Axio Imager Z2.

### DNA-protein interaction- ELISA assay

The CDS of *MtBPC1* was cloned into pGWB6 vector (GFP tag). The CDS of *MtSWN* and *MtSAP18* were cloned into the pGWB18 vector containing a Myc tag. Generated constructs were infiltrated into *N. Benthamiana* leaves and harvested for protein extraction after 48 hours of infiltration. The experiment was performed following the protocol developed by Brand et al. (2010) with a further round of protein incubation. Double-stranded original and mutated fragments of 60-bp oligos (**Supplementary Table S1**) labeled with Biotin were generated according to the same protocol as for the DNA-pull down assay and fixed on Streptavidin coated ELISA plate at 37°C for 1h (4µg). One hour after fixation, the residual spots of the Streptavidin beads were blocked by 5% skimmed-milk, followed by three washings with TBST buffer (100 mM Tris-HCl; 1,5 mM NaCl; pH7,5; 1% Tween 20). Hundred µg of total protein extracted from BPC1 infiltrated leaves were incubated with immobilized biotin labeled double strand for one hour. After washing with TBST, in the second round of protein incubation, MtSWN-Myc or MtSAP18-Myc were incubated for one hour. Anti-Myc tag antibody (HRP) (ab1326) was used at the last round of incubation and processed for detection. Absorbance was measured at 492 nm using 650 nm (plate background) as a reference wavelength in the FLUORO star OMEGA spectrophotometer (BMG LABTECH, Champigny sur Marne, France).

### Chromatin immunoprecipitation and q-PCR assay

The ChIP assay was from Saleh et al., 2008 with modifications. The optimal condition of sonication was P75W, DF26, C/B 200, D 1800, 15 min using a Covaris M220. To enhance the antibody-binding efficiency, the agarose beads used in the protocol of Saleh et al. was replaced by the combination of Protein A magnetic beads and protein G magnetic beads (*Invitrogen*) with a ratio 1:1. H3K27me3 (mAbCam 6002), H3Ac (06–599 EMD Millipore), and GFP (Abcam, ab290) antibodies were used for immunoprecipitation. The nuclear extraction without incubation with antibodies were used as INPUT samples. After immuno-precipitation and washing steps, 30 µl of DNA was used for q-PCR (IP sample). The enrichment was calculated as IP/INPUT (%): 2^(CtINPUT-CtIP)^. The primers for Chip qPCR are shown in **Supplementary Table S1**.

### Seed physiological analyses

To determine seed longevity, mature seeds of R108 and BPC1_OX5 lines were scarified and stored at 75% RH inside the hermetically closed containers with saturated NaCl solution and placed at 35°C. At indicated time points, three replicates of 30 seeds were imbibed in 5 mL water in a 9-cm diameter petri dish with Whatman Filter Paper Grade 1 in the dark, and final germination was measured after 10d. Germination was determined as protrusion of the radicle through the surrounding seed layers. For the ABA sensitivity test, three replicates with 30 mature seeds were scarified and put in 10 µM ABA (mixed isomers, Sigma, St Louis, MO). The imbibed seeds were placed at 20°C, in the dark room. Germination was measured as indicated above.

## Results

### Association analysis identifies *MtABI4* linked to environmental stress and seed longevity

**Figure 1:**
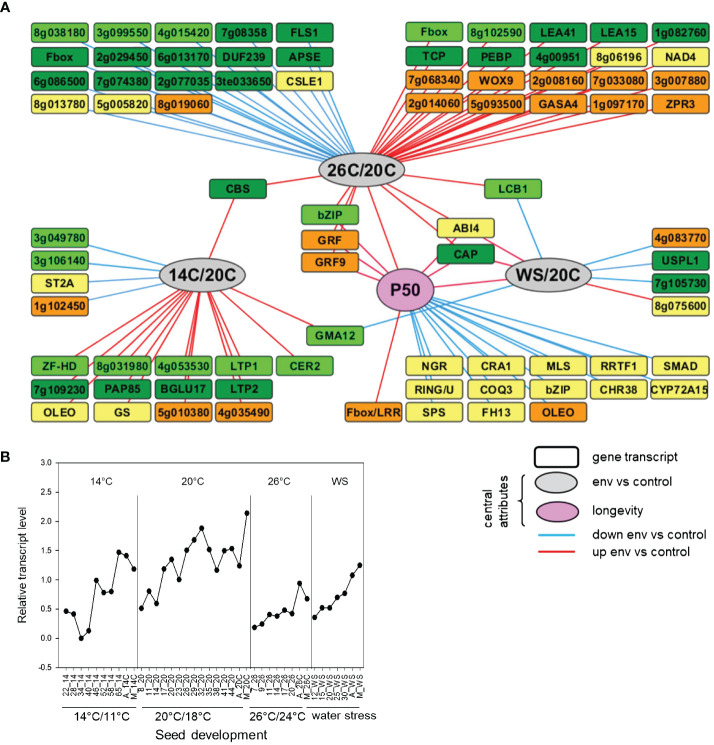
Identification of seed-preferential gene transcripts that are influenced by environmental changes in temperature and water status during seed development in *Medicago truncatula* seeds. **(A)**. Directed network inferred using the RulNet platform and illustrating the use of central attributes. Linkages of seed preferential transcripts (squares) for different phases of seed development with environmental stress conditions compared to control and the acquisition of longevity of seeds (P50) defined as central attributes (circles). The network was exported and enhanced in Cytoscape. Nodes were moved and edges were bundled and reorganized for better readability. Light-green, dark-green, yellow and brown nodes indicate rules discovered with the QNS-I to QNS-IV queries, corresponding to early seed filling, mid seed filling, end of seed filling and maturation drying respectively (**Supplementary Figure S1**). Red and blue edges refer to transcripts decreased or increased by the indicated environment compared to control. Node labels represent gene identifiers of Mtv4.0 or gene symbols when high identity was found for the gene with *Arabidopsis*. Environmental growth conditions correspond to standard conditions (20°C/18°C,16h photoperiod) until flowering followed by the same standard conditions (20C), or high temperature (26C: 26°C/24°C, 16h), low temperature (14C: 14°C/11°C, 16h) or water stress (WS: 20°C/18°C, 16h and maintaining a soil water potential at -0.1 MPa) **(B)**. Expression profiles of *MtABI4* in developing seeds grown in different production environments as described in **(A)**, derived from Righetti et al. (2015). Numbers on the x-axis represent days after pollination. A, abscission; M, mature seed.

When plants of *M. truncatula* are exposed to heat or water stress conditions from flowering onwards, the acquisition of seed longevity is impaired (Righetti et al., 2015). To identify genes that are differentially expressed during seed development in relation to the different environments and longevity (as a proxy for seed vigor), we used the transcriptome dataset published in Righetti et al. (2015) to construct a directed multilevel network with genes that are preferentially expressed in seeds and the longevity value, P50 (i.e. storage time equivalent to 50% loss of viability). The association rule learning algorithm was used with the web-oriented platform RulNet, dedicated to the inference and analysis of regulatory networks from qualitative and quantitative –omics data by means of rule discovery (Vincent et al., 2015). The obtained network (**Figure 1A**) can be considered as a biology-driven clustering visualization rather than as a standard interaction network. As central attributes, we defined the difference in developmental environments (heat-26°C, cold (14°C) and water stress (WS) compared to control conditions (26°C/20°C, 14°C/20°C and WS/20°C) as well as P50, and only rules involving at least one of the central attributes were discovered (**Supplementary Figure S1**). The edges between attributes imply a functional link between the central attributes (environment, longevity) and the other attributes (gene transcript levels) rather than a direct interaction. Overall, the network highlights seed-enriched transcripts whose relative abundance is linked to environmental stress and/or longevity at a particular phase of seed development. The comparison between 26°C and 20°C created the highest number of edges, with higher transcripts at the third phase of development, and lower transcript at final seed maturation (**Figure 1A**). A total of 14 gene transcripts were associated with an increase in longevity in phase 3, including a glyoxysomal *MALATE SYNTHASE*, *LEGUMIN*, and two transcription factors encoding *REDOX RESPONSIVE TRANSCRIPTION FACTOR 1* (RRTF1) and a putative BZIP with closest homology to bZIP62. Two attributes were discovered that were associated with a change in P50 as well as heat- and water-stress environments (**Figure 1A**). One attribute was MtrunA17_Chr8g0373921, encoding CAP, a cysteine-rich secretory protein, antigen 5 protein. The second attribute was MtrunA17_Chr5g0437371, coding for *ABSCISIC ACID INSENSITIVE4*, which was recently shown to be implicated in the regulation of the acquisition of seed vigor traits during seed development of *Medicago* seeds (Zinsmeister et al., 2023). A detailed overview of the expression profiles showed that transcript levels of *MtABI4* were consistently lower throughout seed development when plants were grown under heat and water stress conditions, coinciding with a lower seed vigor, suggesting that upstream regulation of *MtABI4* can affect seed quality acquisition (**Figure 1B**).

### Identification of a BPC class I transcription factor that binds to the *MtABI4* promoter

**Figure 2:**
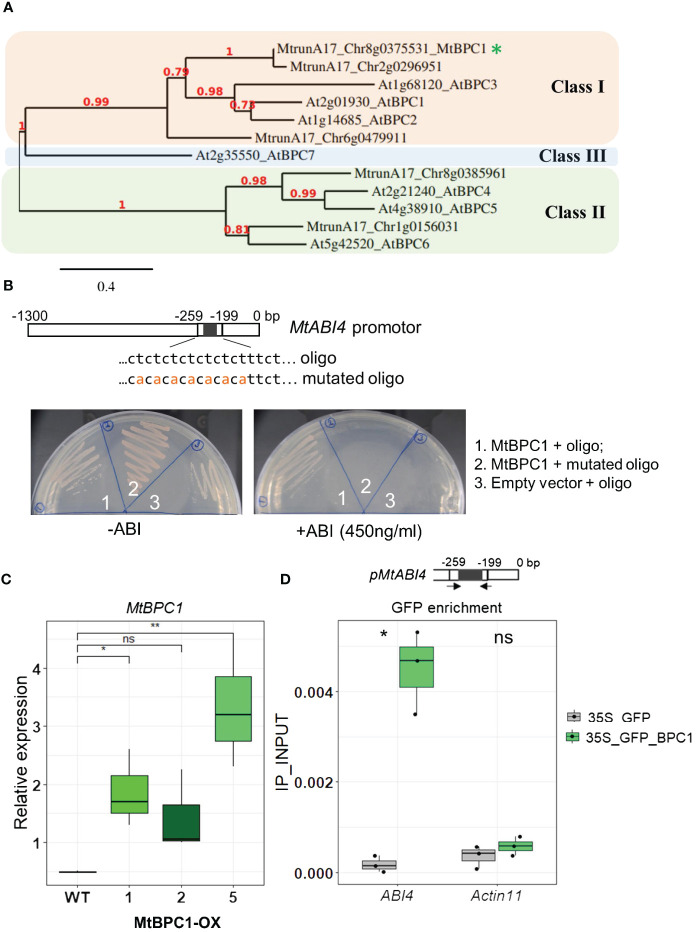
MtBPC1 interacts with *MtABI4* in the promoter region both *in vitro* and *in vivo*. **(A)** Phylogenetic tree of class I, II and III BPC family members from *Arabidopsis thaliana* and *M. truncatula*. The tree was obtained using Phylogeny.fr (Dereeper et al., 2008). The red numbers indicate the boot strap values. The asterisk indicates the *M. truncatula* ortholog of BPC1 identified in this study. **(B)** Yeast one-hybrid assay. The MtBPC1 bait vector was transformed with *MtABI4* oligo (1) and mutated *MtABI4* oligo (2) as baits. (3) empty prey vector transformed with oligo worked as negative controls. **(C)** Relative expression of *BPC1* over-expression lines at mature seeds, normalized by internal controls: *MtAct11* (MtrunA17_Chr2g0278591) and *MtTCTP* (MtrunA17_Chr2g1006185). Data represent three biological replicates (*n* = 45). Asterisks indicates significant difference, two-tailed Student’s t-test, data were compared to the WT, P<0.05. Ns. Not significant **(D)**. Chip-qPCR assay using the GFP antibody. Upper panel shows the position of primers used for RT- qPCR. Lower panel showed GFP enrichment at the BPC1 binding site in the promoter of *MtABI4* in BPC1 overexpression and GFP lines. Seeds are from the second generation. Asterisks indicate significant differences, two-tailed Student’s t-test, P<0.05. Data are represented by three biological replicates (*n* = 15).

To identify the upstream regulators of *MtABI4* during seed development, a Yeast-One Hybrid (Y1H) library screening was performed. A 1289 bp fragment of the *MtABI4* promoter, containing potential binding sites for many regulators as predicted by The Plant Promoter Analysis Navigator (PlantPAN; http://PlantPAN2.itps.ncku.edu.tw), was used as a bait construct (**Supplementary Figure S8**). The prey library was synthesized from the mRNA of seeds harvested at 20, 24, and 28 DAP and at maturity (dry seeds). After screening, nine candidate genes were identified as putative interactors of *MtABI4* (**Supplementary Table S2**). One of them was a GAGA binding motif transcription factor, referred to as BPC protein (MtrunA17_Chr8g0375531). Comparison of the BPC protein homologs with those known for *Arabidopsis* revealed that MtrunA17_Chr8g0375531 is a homolog of *Arabidopsis* BPC class I genes (AtBPC1/2/3) (Monfared et al., 2011) (**Figure 2A**). A blast of Chr8g0375531 sequence with *Arabidopsis* revealed an identity (and e-value) of 54.5% (3e-95), 51.7% (45e-89), and 46.7 (1e-74) with AtBPC1, AtBPC2 and AtBPC3 respectively. Therefore, we named MtrunA17_Chr8g0375531 MtBPC1.

The interaction between MtBPC1 and the promoter of *MtABI4* was tested by individual Y1H transformation using the full coding sequence of MtBPC1 as prey construct and a 60-bp oligo of the promoter region of *MtABI4* that contained the predicted binding site for MtBPC1 (**Figure 2B**). There was a strong interaction between MtBPC1 and the promoter of *MtABI4*, evident from the survival rate of the yeast colonies at very high concentrations of antibiotic selection (**Figure 2B** left panel). This interaction was disrupted when the 60-bp oligo was mutated by substituting nucleotide T to nucleotide A between positions -259 and -199bp of the original promoter sequence (**Figure 2B** right panel).

**Figure 3:**
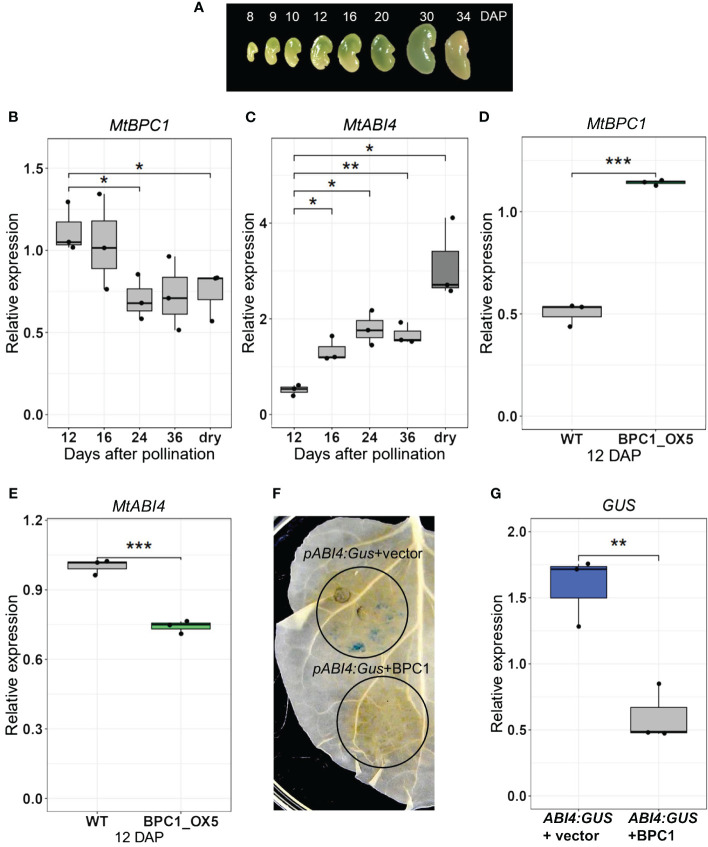
MtBPC1 represses *MtABI4 in planta* and at the early stage of seed development. **(A)** *M. truncatula* seeds at different days after pollination (DAP). Transcript levels of *MtBPC1* **(B)** and *MtABI4* **(C)** at different stages of seed development, normalized by internal controls: *Act11* and *TCTP*. *Data represented three biological replicates (*n* = 15), two-tailed Student’s t-test, data were compared to 12 DAP, *P<0.05*, **P<0.01. Relative expression of *MtBPC1* **(D)** and *MtABI4* **(E)** in 12 DAP seeds of control (empty plasmid) and BPC1 overexpression plants, normalized by internal controls: *Act11* and *TCTP*. **Data represent three biological replicates (*n* = 15). Two-tailed Student’s t-test P<0.01, ***P<0.001, **(F)** MtBPC1 represses the activity of *MtABI4* in *N. Benthamiana* leaves. *MtABI4* promoter-*GUS* co-infiltrated with the pGWB6 empty vector (top panel) or with pGWB6/MtBPC1- (bottom panel). **(G)**. Transcript level of *GUS* normalized by internal controls of *N. Benthamiana* genes *L25* and *EF2*. **Data represent three biological replicates (*n* = 15), two-tailed Student’s t-test. *P<0.01*.

To investigate if the interaction between MtBPC1 and the *MtABI4* promoter also occurs *in vivo* in *Medicago*, stable MtBPC1 over-expression lines (MtBPC1-OX) were generated (**Figure 2C**). Among the three lines, the MtBPC1-OX line 5 (BPC1_OX5) was selected for further analysis due to its strongest increase in *MtBPC1* transcript level (approximately seven times compared with control) (**Figure 2C**) and used to perform ChIP-qPCR using a GFP-antibody to amplify an amplicon surrounding the BPC1-predicted binding site. The expression of *ABI4* was strongly down-regulated by BPC1-OX only at the early stage of seed development (12 DAP), (**Figure 3E**; **Supplementary Figures S2A–C**). Therefore, we used 12 day-old developing seeds of BPC1_OX5 as starting material for the ChIP-qPCR assay. The enrichment of the *MtABI4* promoter containing C-T rich motif was significantly higher in the BPC1_OX5 line than in the control overexpressing GFP alone, thereby confirming the preferable binding site of the BPC1 protein on the promoter region of *MtABI4 in vivo* (**Figure 2D**).

Further confirmation of the interaction was found using an *in vitro* DNA-pull down assay with the *MtABI4* promoter oligo and the mutated form (**Supplementary Figure S3**). Although the *MtABI4* oligo mutant did not completely disrupt the interaction with MtBPC1, probably due to weak non-specific binding of MtBPC1 with Streptavidin beads (**Supplementary Figures S3** **, lane 4**), the band intensity of the *MtABI4* oligo (band 1) was more than three-fold stronger than that of the MtABI4 oligo mutant. The experiment further confirmed the strong binding ability of MtABI4 promoter oligo with the MtBPC1 protein at the CT-rich motif.

### MtBPC1 regulates the repression of *MtABI4* transcription at the early stage of seed development

Considering that MtBPC1 binds to the promoter of *MtABI4*, the next step was to assess whether MtBPC1 activated or repressed the activity of *MtABI4*. Expression analysis of *MtBPC1* at different stages of seed development (**Figure 3A**) revealed that *MtBPC1* and *MtABI4* displayed an opposite expression pattern (**Figures 3B, C**), leading to the hypothesis that MtBPC1 might act as a repressor of *MtABI4* expression. In agreement with this hypothesis, we observed that transcript levels of *MtABI4* in BPC1_OX5 seeds at 12 DAP were reduced compared with seeds generated from the control line transformed with the empty plasmid (**Figures 3D, E**). At 24 DAP and in mature seeds, the expression of *ABI4* was no longer affected, despite *MtBPC1* transcripts remaining upregulated in BPC1_OX5 (**Supplementary Figure S2**). This suggests that MtBPC1 regulates *MtABI4* expression only at the early stage of seed development.

To further ascertain the repressive function of MtBPC1 on *MtABI4*, the 1300 bp promoter of *MtABI4* was fused with the *GUS* sequence and co-infiltrated in *N. Benthamiana* leaves, either with the empty vector or with MtBPC1, both driven by the 35S promoter. GUS staining revealed that the *MtABI4* 1.3kb-promoter was sufficient to induce GUS activity when combined with an empty vector (**Figure 3F**, top panel). In contrast, the leaf area co-infiltrated with MtBPC1 and the *pMtABI4*::*GUS* construct led to a complete absence of GUS staining (**Figure 3F**, bottom panel). In parallel, the transcript level of *GUS* in the leaves was significantly reduced when *pMtABI4::GUS* was co-infiltrated with MtBPC1 compared with co-infiltration with the empty (GFP) vector (**Figure 3G**). These results confirm that MtBPC1 acts as a repressor of *MtABI4* in planta.

Next, we analyzed the transcript level of *MtABI4* at different stages of seed development in three *Tnt1* insertion mutants of MtBPC1 for which the transposon was inserted in the coding sequence of the gene (**Supplementary Figure S4A**). No significant difference in *MtABI4* expression was observed in the *Mtbpc1* mutants compared with the wild type seeds at different developmental stages (**Supplementary Figure S4B**). This result may be due to the redundant roles of MtBPC1 with other MtBPC members, similar to AtBPC class I observed in *Arabidopsis* (Monfared et al., 2011). Based on this result, we did not further investigate the phenotypes of the *Mtbpc1* single mutants, and that a triple mutant *bpc1/bpc2/bpc3* might be needed to overcome genetic redundancy (Simonini and Kater, 2014).

### *MtBPC1-OX* and *Mtabi4* mutant seeds show comparable deregulation of genes related to photosynthesis and hormone responses at early seed development

**Figure 4:**
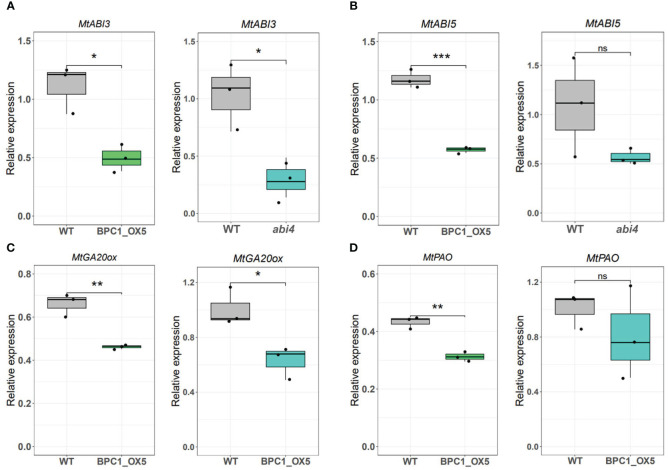
Relative expression of putative target genes of MtABI4 in MtBPC1-OX seeds at 13 DAP. Transcript levels of *MtABI3* **(A)**, *MtABI5* **(B)**, *MtGA20ox* **(C)**, *MtPAO* **(D)** genes in WT transformed with empty plasmid and BPC1_OX5 seeds, and *Mtabi4–1* seeds and associated wildtype, obtained by RT-qPCR. The relative expression was normalized by two internal controls: *Act11* and *TCTP*. *Data are represented by three biological replicates (*n* = 15), two-tailed Student’s t-test. P<0.05, **P<0.01, ***P<0.001, ns, Not significant.

Considering the suppressive action of MtBPC1 on *MtABI4*, we investigated if misregulated genes in seeds from *Mtabi4* mutants (Zinsmeister et al., 2023) are also misregulated in seeds of the MtBPC1-OX5 line with those. Since an RNAseq dataset was already available for *Mtabi4* seeds harvested at 13 DAP (Zinsmeister et al., 2023), we identified 7 key genes from these data. Plants of both the Mt*abi4* mutant and BPC-OX5 line were grown together with their wildtypes and seeds were harvested at 13 DAP. Transcript levels of genes involved in ABA or GA signaling and metabolism, such as *ABI3*, *ABI5*, and *GA20ox* were reduced in both the *abi4* mutant and BPC1_OX5 compared to their controls (**Figures 4A–C**). Likewise, RT-qPCR showed a reduction of the transcripts of pheide a oxygenase (*PAO*) in the BPC1_OX5 seeds (**Figure 4D**).

Next, we investigated if the *Mtabi4* phenotypes were also visible in the MtBPC1-OX seeds, such as speed of germination or longevity (Zinsmeister et al., 2023). However, no difference was found in the speed of aging (**Supplementary Figure S5A**) or speed of germination in the presence of ABA between the BPC1-OX line and control seeds (**Supplementary Figure S5B**). The absence of those phenotypes in MtBPC1-OX could be explained by the effect of BPC1 on the expression of *ABI4* only at the early seed stage, but not at the mature seed stage, when seed germination and longevity are acquired (**Figures 3D, E**; **Supplementary Figure S2**).

### Repression of *MtABI4* transcription is associated with H3K27me3 deposition via binding of MtBPC1 to a subunit of the PRC2 complex

**Figure 5:**
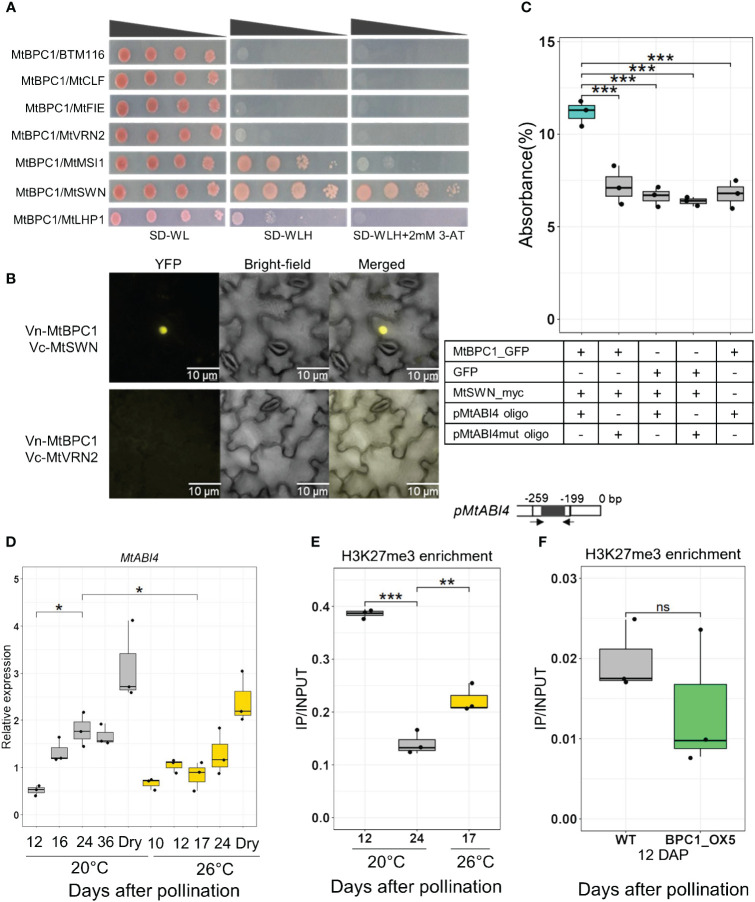
MtBPC1 interacts with MtSWN to suppress *MtABI4* transcription via the modification of H3K27me3 level. **(A)** Yeast two-hybrid assay. Successfully transformed yeast on the medium SD-WL were diluted 1x, 10x, 100x, and 1000x and spread on SD-WLH and SD-WLH+ 3mM 3-AT. Empty vector BTM116 and empty vector Pvp16 were used as negative controls. **(B)** Bimolecular Fluorescence Complementation (Bi-FC) assay for the interaction between MtBPC1 and MtSWN. Vn, Venus N-terminal; Vc, Venus C-terminal. MtVRN2 was used as negative control. **(C)** DPI-ELISA assay for the formation of a MtBPC1/MtSWN/*pMtABI4* complex. The interaction was measured by the percentage of absorbance. Data are represented by three biological replicates (*n* = 9). mut, mutated. ***P<0.001 two-tailed Student’s t-test. **(D)** Transcript levels of *MtABI4* at different stages of seed development at 20°C and 26°C. *Act11* and *TCTP* were used as internal controls. Dry, mature dry seeds. Data represent three biological replicates (*n* = 15). Asterisk indicates the significant difference between samples at 12 DAP and 24 DAP or 24 DAP and 17 DAP, 26°C, *P<0.05 using a two-tailed Student’s t-test. **(E)** H3K27me3 enrichment at the promoter of *MtABI4* after immuno-precipitation by H3K27me3 antibody, measured by the ratio IP/INPUT. **P<0.01, ***P<0.001, two-tailed Student’s t-test. **(F)** H3K27me3 enrichment of WT and MtBPC1_OX5 in the promoter of *MtABI4* at 12 DAP measured by IP/INPUT. Data represent three biological replicates (*n* = 15). Ns, not significant, two-tailed Student’s t-test.

In *Arabidopsis*, AtBPC1 and BPCs class II proteins (AtBPC4 and AtBPC6) can repress the activity of *AtABI4* in roots by recruiting SWINGER (SWN), a subunit of the PRC2 complex, to the *AtABI4* promoter to modify H3K27me3 levels (Mu et al., 2017b). AtBPC6 was also shown to interact with LHP1, a subunit of the PRC1 complex (Hecker et al., 2015). Thus, we tested the hypothesis that MtBPC1 interacts with SWN and LHP1 in *Medicago*. Both candidates were cloned together with several other subunits of the *Medicago* PRC2 complex: MtCLF, MtFIE, MtVRN2 and MtMSI1 and analyzed by Yeast-two-Hybrid assay (Y2H) (**Figure 5A**). MtBPC1 showed a strong interaction with MtSWN (**Figure 5A**). Only a weak interaction with MtLHP1 was found, possibly because the homology between MtBPC1 and AtBPC6 was mainly restricted to the conserved region (52% identity, e-value = 4e-39). In addition, a weak interaction was detected between BPC1 and MSI1. Considering the strong interaction between BPC1 and SWN, we focused on this subunit for subsequent studies. To further confirm the interaction between BPC1 and SWN, a Bimolecular Fluorescence Complementation (Bi-FC) assay was performed with the transient expression of both Vc-MtBPC1 and Vn-MtSWN proteins in *N. Benthamiana* leaves. A strong YFP signal was detected in the nucleus of the leaves co-infiltrated with BPC1 and SWN 24 h after infiltration (**Figure 5B**; **Supplementary Figure S9**), whereas the sample co-infiltrated by Vc-BPC1 and Vn-VRN2 (a control protein that shows no interaction with BPC1 in the Y2H assay, **Figure 5A**) did not show any signal (**Figure 5B**).

Next, we investigated if MtBPC1 only binds to MtSWN or whether they interact as a complex within the *MtABI4* promoter using a DNA-Protein Interaction ELISA (DPI-ELISA) assay (Brand et al., 2010; Hecker et al., 2015). Immobilized double strand of *ABI4* promoter was incubated with BPC1-GFP and subsequently with SWN-myc fusion proteins. The recruitment of SWN to the promoter of *ABI4* by BPC1 was detected by a Myc-tag antibody and quantified by relative luminescence. Only the reaction with all three components (MtBPC1/MtSWN/*MtABI4* promoter) exhibited a strong absorbance value, whereas the reactions containing the mutated *MtABI4* promoter or lacking one of the proteins displayed only background absorbance (**Figure 5C**). These results confirmed that MtBPC1 functions as a binding partner for SWN to target *MtABI4* promoter.

Since MtBPC1 recruits the PRC2 complex to the promoter of *MtABI4* via interaction with MtSWN, and the PRC2 complex is known to regulate the activity of the target genes via the modification of H3K27me3 levels, we next investigated the link between the transcription levels of *MtABI4* and H3K27me3 enrichment in its promoter. First, the expression of *MtABI4* was checked at different time points of seed development in the R108 genotype grown under standard condition (20°C) and under heat stress (26°C), which was shown to lead to a decrease in transcript level in the Jemalong genotype (**Figure 1B**). The transcript level of *MtABI4* increased significantly between 12 DAP and 24 DAP when grown under standard condition, as found previously (**Figure 5D**). Under continuous heat, seed development was accelerated, with seeds at 17 DAP grown at 26°C corresponding to the same stage as seeds at 24 DAP at 20°C. The heat led to a significant reduction in *MtABI4* transcript levels between these comparable developmental stages, confirming the data obtained for the Jemalong genotype (**Figure 1B**). The enrichment of H3K27me3 in seeds grown at 20°C was higher at the early developmental stage (12 DAP) and decreased upon further development (24 DAP), concomitant with the increase in *MtABI4* transcript level (**Figure 5E**). In addition, enrichment of H3K27me3 was higher during heat stress at comparable developmental stage (**Figure 5E**), correlating *MtABI4* expression to H3K27me3 enrichment on its promoter. Transcript levels of the *MtSWN* subunit increased with the repression of *MtABI4* expression during seed development (**Supplementary Figure S6A**). There was no significant difference in H3K27me3 deposition between the *bpc1–1 Tnt1*-mutant and wild type seeds, probably due to the redundant role of *MtBPC* genes in controlling *MtABI4* expression (**Supplementary Figure S4C**). Since *MtABI4* expression was significantly reduced in BPC1-OX seeds at 12 DAP (**Figure 3E**), H3K27me3 levels at the *MtABI4* promoter were assessed in the BPC1-OX seeds at 12 DAP. However, no significant difference could be detected in H3K27me3 enrichment between BPC1-OX seeds and control seeds (**Figure 5F**). From this result, we speculated that there might be an additional regulatory mechanism of *MtABI4* transcription alongside H3K27me3 at the early stages of seed development.

### Transcription of *MtABI4* is correlated with H3ac levels via the interaction of Sin3- associated peptide 18 (SAP18) with MtBPC1

**Figure 6:**
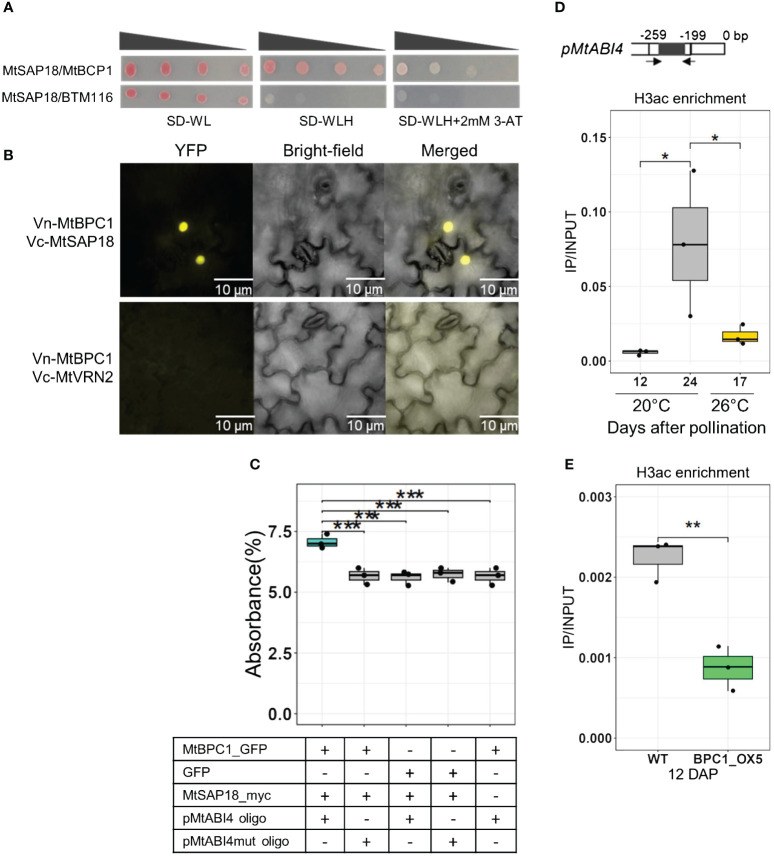
MtBPC1 interacts with MtSAP18 to suppress *MtABI4* transcription via the modification of H3ac. **(A)** Yeast two-hybrid assay. Successfully transformed yeast on the medium SD-WL were diluted 1x, 10x, 100x, and 1000x and spread on SD-WLH and SD-WLH+ 3mM 3-AT. Empty vector BTM116 and empty vector Pvp16 were used as negative controls. **(B)** Bimolecular Fluorescence Complementation (Bi-FC) assay for the interaction between MtBPC1 and MtSAP18. Vn, Venus N-terminal; Vc, Venus C-terminal. MtVRN2 was used as negative control, and is a duplication from **Figure 5B**. **(C)** DPI-ELISA assay for the formation of MtBPC1/MtSAP18/*pMtABI4* complex. The interaction was measured by percentage of absorbance. Data represent three biological replicates (*n* = 9). Asterisk indicates significant difference, two-tailed Student’s t-test, P<0.01, compared to the MtBPC1/MtSAP18/*pMtABI4* oligo sample. mut, mutated. **(D)** H3ac enrichment at the promoter region of *MtABI4* from wild type R108 seeds at 20°C and 26°C measured by the ratio IP/INPUT. *P<0.05, two-tailed Student’s t-test. **(E)** H3ac enrichment at the promoter of *MtABI4* from WT and MtBPC1-OX seeds at 12 DAP. Data represented three biological replicates (*n* = 15). **P<0.01 two-tailed Student’s t-test. DAP, days after pollination.

In *Drosophila*, a GAGA transcription factor is known to interact with Sin3 associated polypeptide 18 (SAP18), a subunit of the Sin3-deacetylation complex, and suppresses the transcript level of target genes via de-acetylation (Espinás et al., 2000). To investigate if a similar regulation occurs in *Medicago*, a unique homolog of SAP18 was identified (MtrunA17_Chr8g0351071) and cloned into corresponding vectors for further Y2H and Bi-FC analyses. Y2H analysis revealed a strong interaction between MtBPC1 and MtSAP18 (**Figure 6A**). Likewise, the Bi-FC assay showed a strong YFP signal in the presence of both BPC1/SAP18 but not for the negative control Vc-BPC1 and Vn-VRN2 (**Figure 6B** **; (** **Supplementary Figure S9**), further confirming the interaction between MtBPC1 and MtSAP18. The DPI-ELISA assay showed that the MtBPC1-MtSAP18 binds to the *MtABI4* promoter *in vitro*, evident from the high percentage of absorbance compared to reactions that lack one of the components of the complex (**Figure 6C**).

Since SAP18 controls the expression of target genes by changing acetylation levels (De Ruijter et al., 2003), we examined H3ac enrichment in the *MtABI4* promoter in the three samples that showed a significant difference in *MtABI4* transcript level: 12DAP seeds produced at 20°C, 24 DAP seeds at 20°C, and 17 DAP seeds at 26°C. During seed development, the enrichment of H3ac was very low at 12 DAP and increased strongly at 24 DAP, when *MtABI4* transcript levels increased (**Figure 6D**). Upon exposure to heat stress, H3ac enrichment at 17 DAP is significantly reduced compared with 24 DAP seed at 20°C, corresponding to the decrease in *MtABI4* transcript level, demonstrating a strong correlation between *ABI4* expression and H3ac enrichment (**Figure 6D**). In addition, a significant difference in H3ac enrichment was observed between seeds from the BPC1-OX and control lines, coinciding with the reduced expression of *MtABI4* in the BPC1-OX seeds at 12 DAP (**Figure 6E**). Similar to H3K27me3, there was no significant difference in H3ac enrichment between WT and *bpc1–1 Tnt1* mutant (**Supplementary Figure S4D**).

Transcript levels of *MtSAP18* during seed development only reduced slightly when plants were grown at 20°C. However, growing plants at 26°C resulted in a strong increase in *MtSAP18* transcript level in 17-day old seeds (**Supplementary Figure S6B**). This induction of MtSAP18 under heat stress corresponded to a low H3ac level in the promoter of *ABI4*, resulting in delayed activation, suggesting its functions as a heat stress-induced factor acting during the second part of seed maturation.

### MtBPC1 protein interacts with MtSWN and MtSAP18 at different domains

In *Arabidopsis*, BPC class I, including AtBPC1, AtBPC2, and AtBPC3, do not have any significant similarity in sequence or predicted motifs with the previously identified protein structure, except for the Zinc-DNA binding domain at the C-terminal end (Meister et al., 2004). On the other hand, the structure of BPC class II, which comprises BPC2, BPC4, and BPC6, has been well identified with three domains: Coil-Coiled domain (CC), Nuclear Localization Signal (NLS) and Zinc-finger like DNA-binding domain (Zn) (Wanke et al., 2011). The CC domain was shown to be required for the dimerization of BPC proteins (Wanke et al., 2011) and for the interaction between BPC6 and LHP1 (Hecker et al., 2015).

**Figure 7:**
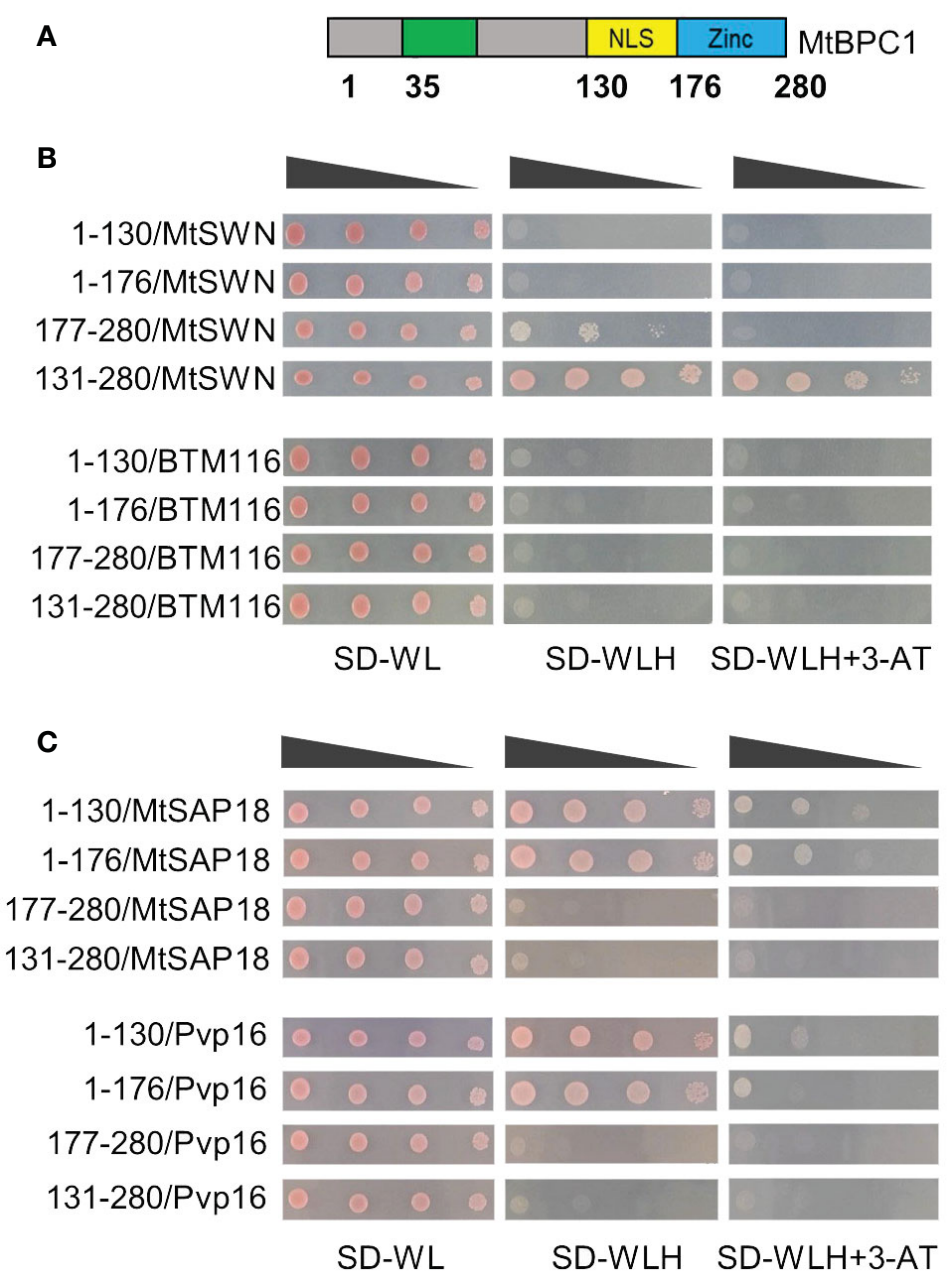
BPC1 interacts with SWN and SAP18 at different domains. **(A)** Schematic diagram of BPC1 protein and fragments used for Yeast two-hybrid assay. NLS. Nuclear localization signal. Zn. zinc-finger like DNA-binding domain. The number indicates the positions of amino acid. **(B)** The interaction between different fragments of BPC1 with SWN. Successfully transformed yeast on the medium SD-WL were diluted 1x, 10x, 100x, and 1000x and spread on SD-WLH and SD-WLH+ 3 mM 3-AT. Empty BTM116 served as negative control. **(C)** The interaction between different fragments of BPC1 with SAP18. Successfully transformed yeast on the medium SD-WL were diluted 1x, 10x, 100x, and 1000x and spread on SD-WLH and SD-WLH+3-AT. Empty Pvp16 vector served as negative control.

To better understand the function of the different regions of the MtBPC1 protein, we aligned its protein sequence to AtBPC1, AtBPC4, and AtBPC6 (**Supplementary Figure S7**). Similar to BPC class I in *Arabidopsis*, there was a large variation in the sequences corresponding to the positions of NLS and CC domain in AtBPC4/AtBPC6 with *Medicago* MtBPC1 (**Supplementary Figure S7**), except for the conserved Zn domain. To better understand which part of MtBPC1 is responsible for protein-protein interactions, we generated different fragments of MtBPC1 based on the position of amino acid corresponding to different domains of AtBPC6 and examined their interaction with MtSWN and MtSAP18 by Y2H (**Figure 7A**). Interestingly, the fragment corresponding to the CC domain position (1- 130) or the fragment corresponding to the CC domain and the NLS positions (1–176) did not show any interaction with MtSWN (**Figures 7A, B**). However, the fragment corresponding to the NLS position and Zn domain (131–280) was sufficient for the interaction with SWN (**Figure 6B**) *in vitro*.

A similar study was performed with the MtSAP18 protein. In contrast to interaction with SWN, the N-terminal part of MtBPC1 was responsible for the interaction with MtSAP18 (**Figure 7C**). On the other hand, the fragment corresponding to NLS and Zn domain (131–280) did not show any interaction with MtSAP18 (**Figure 7C**). These data suggest that MtSWN and MtSAP18 bind with MtBPC1 via different protein domains.

## Discussion

### Regulation of *MtABI4* by the combined action of the PRC2 and Sin3-deacetylation complex

**Figure 8:**
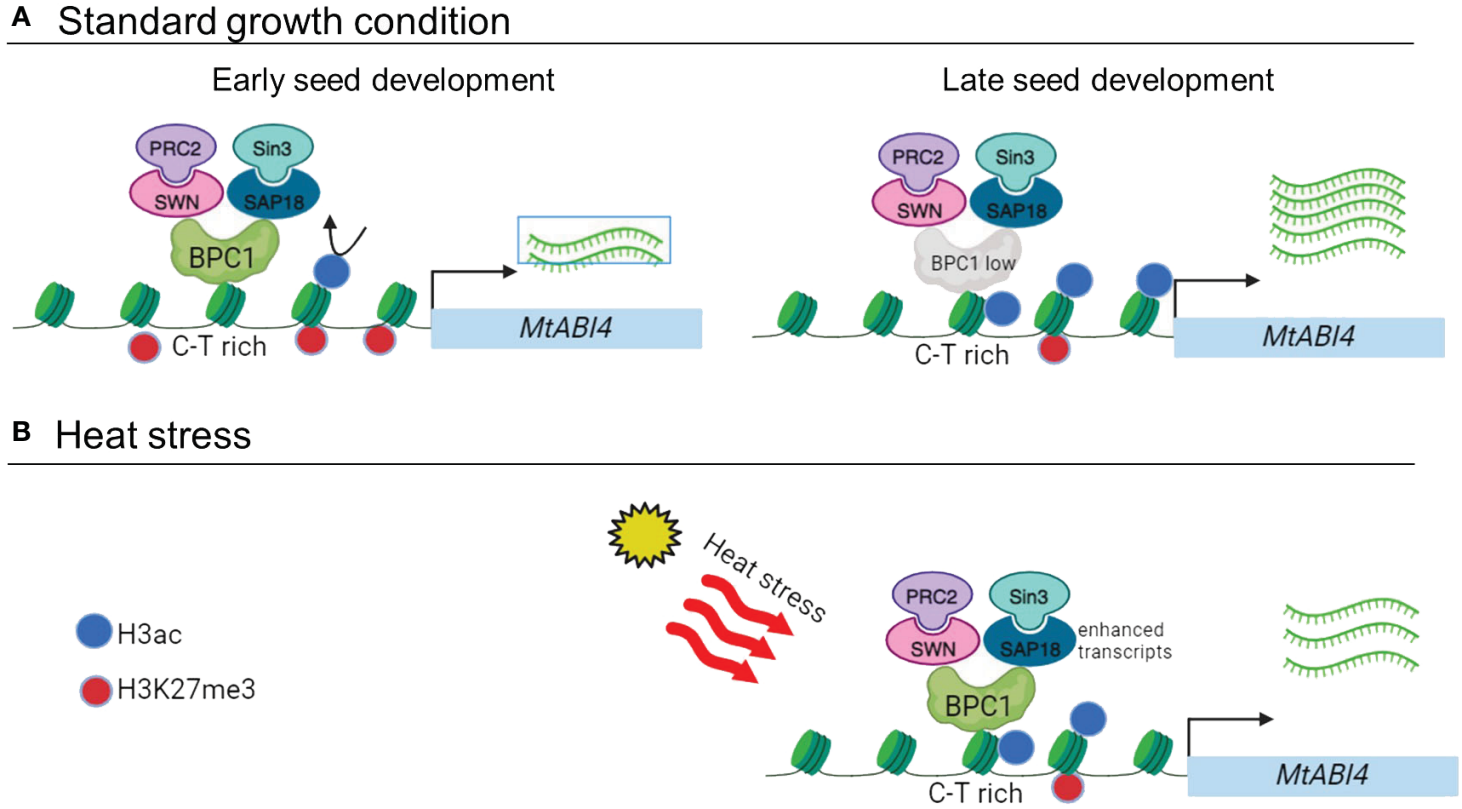
Proposed working model **(A)**. At the early stage of seed development (left panel, 8–12 DAP), the MtBPC1 transcription factor binds to the promoter of *MtABI4* at the CT rich region. MtBPC1 recruits MtSWN, a subunit of the PRC2 complex, which has methyl-transferase activity, to the promoter of *MtABI4* and represses *MtABI4* transcription by changing the H3K27me3 level. In parallel, MtBPC1 interacts with MtSAP18, a sub-unit of the Sin3 complex, and represses the transcript level of *MtABI4* by modification of H3ac. At the later stage of seed development (right panel, > 24 DAP), when the transcript level of *MtBPC1*, *MtSWN* and *MtSAP18* is reduced, the expression of *MtABI4* is derepressed. **(B)** Under heat stress condition, the accumulation of both H3ac and H3K27me3 in the mature seed stage of *MtABI4* (17 DAP) was reduced, resulting in delayed gene activation. Created with BioRender.com.

The expression of *ABI4* is relatively low during the vegetative stage and induced in developing and imbibing seeds (Söderman et al., 2000). A high expression of *ABI4* is known to have harmful effects, such as a decrease in plant height and reduced seed production in adult plants (Shu et al., 2016). Consistently, most of the identified regulators of *ABI4* have a negative effect on its transcript level. In this study, we identified MtBPC1, a transcription factor that binds directly to the *ABI4* promoter via the CT-rich motif and represses transcription of *MtABI4* in developing seeds. This mechanism in *Medicago* seeds is comparable to that found in *Arabidopsis* roots (Mu et al., 2017b). We discovered that MtBPC1 interacts with MtSWN and represses the transcription of *MtABI4* in *Medicago* seeds via the deposition of H3K27me3, a well-known histone mark catalyzed by the PRC2 complex. Interestingly, besides MtSWN, we identified another interactor of MtBPC1, MtSAP18, which is a member of the Sin3-deacetylation complex that represses gene transcription by H3ac deposition. The combination of H3K27me3 and H3-deacetylation appears sufficient to repress *MtABI4* transcription at the early stage of seed development and during heat stress (**Figure 8**). The first *in vivo* interaction of plant SAP18 with PRC complexes was found by Qüesta et al. (2016) and further validated by Mikulski et al. (2022). Two recent reports reveal that the combination of PRC2 and Sin3-deacetylation complexes is sufficient for long-lasting gene repression (Baile et al., 2021; Lin et al., 2022). Baile et al. (2021) discovered that the Ethylene-responsive element binding factor-associated Amphiphilic Repression (EAR) domain-containing transcription domain connects H3K27me3-mediated PRC2 with H3ac deacetylation, probably via SAP18 or TPL. Lin et al. (2022) showed that P55, a newly identified component of the PRC2 complex that plays an essential role in H3K27me3 distribution, is associated with Sin3 to maintain H3K27me3 levels in *M. oryzae*. Generation of single and double mutants of SWN and SAP18 will be needed to address the question of whether both proteins work independently or cooperatively for gene repression based on corresponding histone marks. Besides SWN, a weak interaction between BPC1 and MSI1 was detected by Y-2H (**Figure 5A**). MSI1 is known to co-purify with HDA6 and HAD19, two members of the Sin3-deacetylation complex to lower the level of H3K9ac and to increase H3K27me accumulation on flowering genes (Xu et al., 2022) and ABA response genes (Mehdi et al., 2016).

Interestingly, under heat stress conditions, the transcript level of *MtSAP18* at 17 DAP was significantly higher compared to standard conditions, consistent with low levels of H3ac deposition and *ABI4* repression (**Figure 6**; **Supplementary Figure 6B**). MtSAP18 is a single copy gene, highly conserved between plants and animals, and globally expressed in all organs. Yet, in *Arabidopsis*, the *sap18* mutant does not exhibit any obvious phenotype until the plants are challenged by salt and drought stress (Song and Galbraith, 2006). Considering the high induction of *MtSAP18* under heat stress in our study, we speculate that MtSAP18 might function as a mobile sensor to sense the stress signals and bridge the Sin3 deacetylation complex to the chromatin of target genes.

### *MtABI4* repression by MtBPC1 is limited to early developmental stages

Our study suggests that MtBPC1 represses *MtABI4* expression only at the early stages of seed development, at the transition between the end of embryogenesis and the start of seed maturation. At this early stage, the BPC transcription factor recruits SWN and SAP18 to the promoter of *ABI4* to repress its transcription level via modifying two histone marks: H3K27me3 and H3ac. When the seeds enter the mature stage, *ABI4* transcription is no longer repressed due to the reduced expression of BPC, SWN, and SAP18. The repression at the early stage is essential since inappropriate early activation of *ABI4* might lead to chloroplast dedifferentiation, which normally starts at 24 DAP (Zinsmeister et al., 2023). Later during seed maturation, overexpression of *MtBPC1* no longer affected the expression of *ABI4*, despite *MtBPC1* transcripts remaining upregulated in the BPC1-OX line (**Supplementary Figures S2C, D**). Consistent with this timely regulation, no clear difference in phenotypes was evident in mature seeds of wild type and BPC1-OX line. Possibly, increasing the level of *BPC1* alone is not sufficient to modulate *ABI4* transcription, possibly because other co-regulators, such as SWN and SAP18, whose transcript levels decrease upon further maturation, are missing. Alternatively, the use of the 35S promoter might not have been optimal to induce large differences in *BPC1* expression, since it is known not to function well during early embryogenesis (Sunilkumar et al., 2002). Interestingly, we found that the repression of *ABI4* at 12 DAP in the BPC1-OX line can be explained mainly by a change in H3ac rather than H3K27me3, suggesting that the Sin3 de-acetylation complex might have a dominant role over PRC2 in controlling *ABI4* expression at this early stage. Since we found a similar CT-rich motif of *ABI4* in other legumes (data not shown), it appears that the binding motif is conserved. Thus, the regulation module *ABI4* pro-BPC1-SWN/SAP18 might exist in other legumes and play a key role in controlling *ABI4* expression in seed development under both standard and stressful growth conditions (**Figure 8**). Future studies are needed to investigate the dynamics of the module *ABI4-*pro-BPC1-SWN and *ABI4-*pro-BPC1-SAP18 at different developmental stages and stress conditions.

### The role of BPC proteins in the regulation of *MtABI4* and other downstream targets

In *Arabidopsis*, several BPC proteins were found to interact with the *ABI4* promoter using Y1H screening (Mu et al., 2017b). Although the tissue origin of the protein library was not specified, it was demonstrated that BPCs and *ABI4* coordinate their activities to fine-tune the levels of PIN-FORMED1 (PIN1), a component of the auxin signaling pathway and modulate lateral root formation (Mu et al., 2017b). Here, we used a protein library from different stages of seed development from early to mature seeds, and only MtBPC1 was identified as an interactor of the 1.3kb promoter of *MtABI4*.

The genome of *Medicago* contains 5 BPC proteins, compared to seven in *Arabidopsis*. One *Medicago* gene (MtrunA17_Chr2g0296951) is a truncated protein that has a high similarity to MtBPC1 (MtrunA17_Chr8g0375531) (**Figure 2A**). Compared to *Arabidopsis*, no Class III BPC protein exists, and whereas for both species, the Class I and Class II BPC proteins group together, the *Medicago* BPC Class I members separate from the *Arabidopsis* Class I members (**Figure 2A**). Interestingly, *MtBPC1* and *MtBPC4* (MtrunA17_8g0385961) show very similar expression patterns in seed development (https://lipmbrowsers.toulouse.inra.fr/pub/expressionAtlas/app/v2), raising the intriguing question whether both MtBPC1 and MtBPC4 regulate *MtABI4*, although MtBPC4 was not detected in our Y1H screen. Since the N terminal part (1- 130) and the middle domain of MtBPC1 (130 - 176) do not share any sequence similarity or conserved motif compared with the previously identified proteins, it is challenging to predict the specific function for each domain. Here, we identified that MtBPC1 interacts with other proteins via different amino acid positions, as such enhancing the number of the regulators able to interact with MtBPC1. On the other hand, the motif sequence responsible for the localization of MtBPC1 and the importance of its localization for gene regulatory mechanism in seed development needs to be addressed in future studies.

Besides *MtABI4*, MtBPC1 might regulate other downstream factors during seed development. In *Arabidopsis*, BPC1 binds directly to the GA-rich consensus sequence of SEEDSTICK (*STK*) promoter exclusively expressed in ovules, facilitating the binding affinity of *STK* with co-repressor complex AP1-SVP-SEU-LUG (Simonini et al., 2012). BPCs class I are direct regulators of the HOMEOBOX genes SHOOTMERISTEMLESS (*STM*) and BREVIPEDICELLUS/KNAT1 (*BP*), responsible for the regulation of meristem size (Simonini et al., 2012). *FUS3*, a gene known to regulate the developmental phase transition in *Arabidopsis*, is controlled by class I BPCs (BPC1–3) by recruiting the FIS-PRC2 complex. The ectopic expression of *FUS3* in *bpc1–3* mutant causes delayed or arrested embryo development (Wu et al., 2020). Cucumber CsBPC1 and CsBPC3 bind to the promoter of *ABI3* and suppress its expression (Mu et al., 2017a). AtBPC class I proteins also target *LEAFY COTYLEDON 2* (*LEC2*), a master regulatory of seed development, and function by repressing *LEC2* in the vegetative stage and releasing its expression in the embryo seed via the modification of H3K27me3 by PRC2 complex (Berger et al., 2011). We show that in the MtBPC1_OX5, *ABI3* and *ABI5* were repressed at the early stage of seed development. This could be because a change in *MtABI4* level modulates them (Zinsmeister et al., 2023), but since the CT-rich motif is present in their promoter, they could also be direct targets of the MtBPC1-PRC2 and/or MtBPC1-Sin3 complexes.

## Data availability statement

The original contributions presented in the study are included in the article/**Supplementary Material**. Further inquiries can be directed to the corresponding authors.

## Author contributions

TD: Conceptualization, Investigation, Methodology, Validation, Visualization, Writing – original draft, Formal analysis. DL: Investigation, Writing – original draft. JLV: Investigation, Writing – original draft. BLV: Investigation, Writing – original draft. JD: Data curation, Writing – review & editing. JV: Writing – review & editing. OL: Writing – review & editing, Funding acquisition, Project administration. JB: Conceptualization, Investigation, Project administration, Supervision, Writing – review & editing.

## Supplementary material

Supplementary Figure 1 Developmental stages **(A)** and rules **(B)** used for queries in RulNet.

Supplementary Figure 2 Expression of *MtBPC1* and *MtABI4* in BPC1-OX lines during seed development.

Supplementary Figure 3 DNA-pull down assay of the MtABI4 promoter fragment by MtBPC1.

Supplementary Figure 4 Transcript level and histone mark modification of *MtABI4* in *bpc1 Tnt* insertion mutants.

Supplementary Figure 5 Physiological characterization of MtBPC-OX seeds.

Supplementary Figure 6 Relative expression of MtSWN **(A)** and MtSAP18 at 20°C and 26°C by RT-qPCR.

Supplementary Figure 7 Protein alignment of AtBPC1, AtBPC4, AtBPC6 and MtBPC1.

Supplementary Figure 8 The promoter sequence of *MtABI4* used as a bait construct for Yeast One Hybrid library screening.

Supplementary Figure 9 Bi-FC of the interaction between BPC1-SWN **(A)** and BPC1-SAP18 **(B)**.

Supplementary Table 2 Regulators of *MtABI4* identified from Yeast one-hybrid library screening.
