# Supplementary material for: BASIC PENTACYSTEINE1 regulates ABI4 by modification of two histone marks H3K27me3 and H3ac during early seed development of Medicago truncatula
Source: Front Plant Sci. 2024 Jun 10;15:1395379. doi: 10.3389/fpls.2024.1395379 (PMC11194320; doi:10.3389/fpls.2024.1395379)
Supplement: Supplementary Figure 1 — Developmental stages (A) and rules (B) used for queries in RulNet. [file Presentation_1.pdf]

A

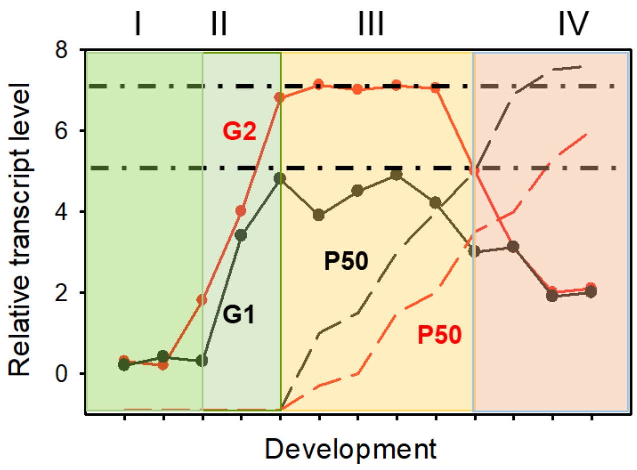

Scheme indicating the determination of the different developmental phases of seed development of *Medicago truncatula* used in the query

B

Queries used in RULNET:

<u>QSN-I</u>
FINDRULES
SCOPE t1 IN SSP,
t2 IN SSP
WHERE t1.stress != t2.stress AND t1.DEVSTAGE = 1 AND t2. DEVSTAGE = 1
HAVING t1.ATT - t2.ATT > 2 OVER ALL
AND 20C/14C: t1.ATT = '20' AND t2.ATT = '14' OVER stress
AND 20C/WS: t1.ATT = '20' AND t2.ATT = 'WS' OVER stress
AND 20C/26C: t1.ATT = '20' AND t2.ATT = '26' OVER stress

<u>QSN-II</u>
As QSN1 but
t1.DEVSTAGE = 2 AND t2. DEVSTAGE = 2

<u>QSN-III</u>
As QSN1 but
t1.DEVSTAGE = 3 AND t2. DEVSTAGE = 3

<u>QSN-IV</u>
As QSN1 but
t1.DEVSTAGE = 4 AND t2. DEVSTAGE = 4

Supplementary Figure S1

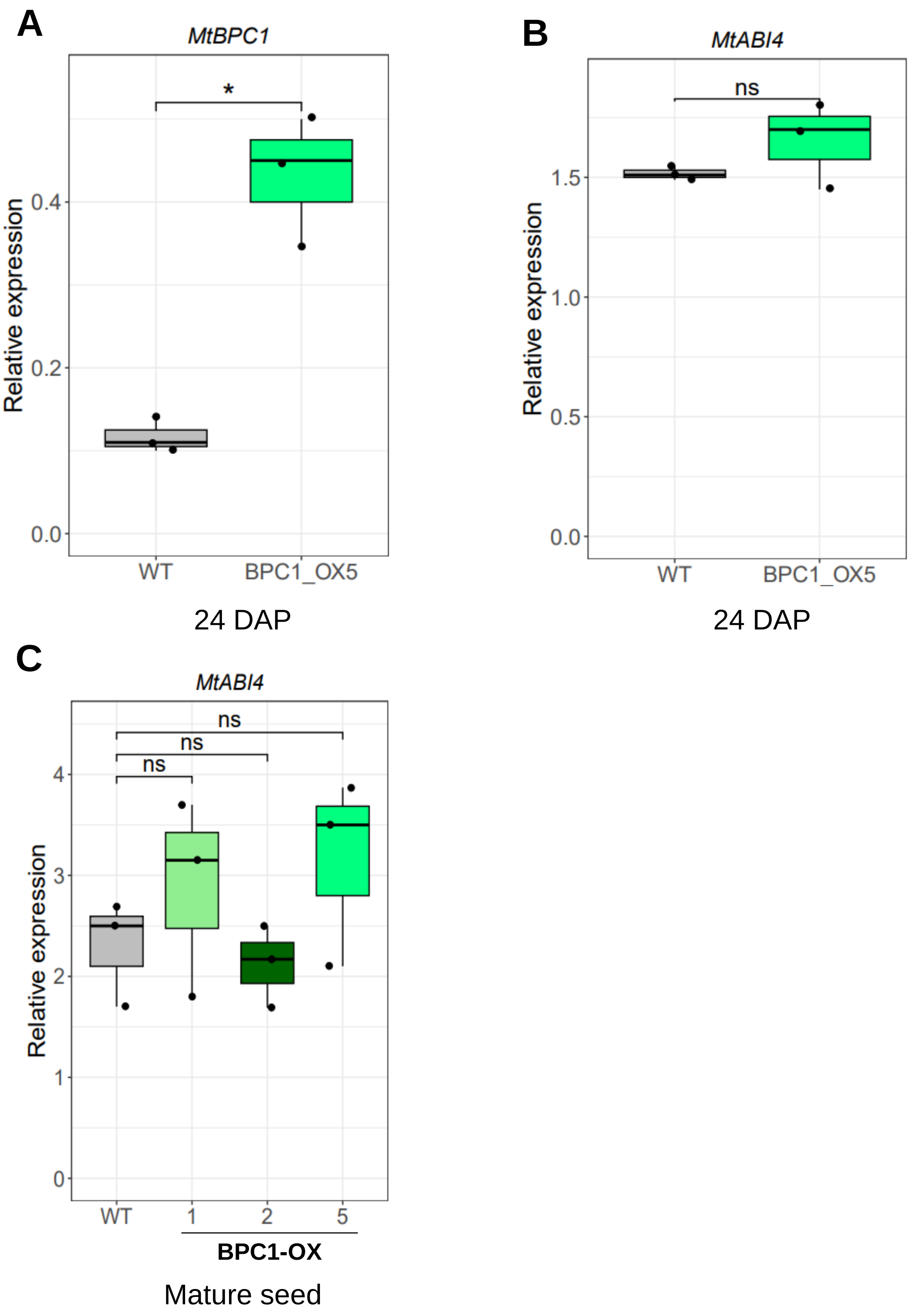

Supplementary Figure S2. Relative expression of *MtBPC1* and *MtABI4* in BPC1-OX lines at 24 DAP (A and B) and mature seed (C). *. *P<0.01*, ns. Not significant, *P>0.05,* stutent t.test

BPC1-GFP	+	-	+	+
GFP	-	+	-	-
biotinylated oligo	+	+	-	-
non-biotinylated oligo	-	-	-	+
mutated biotin oligo	-	-	+	-

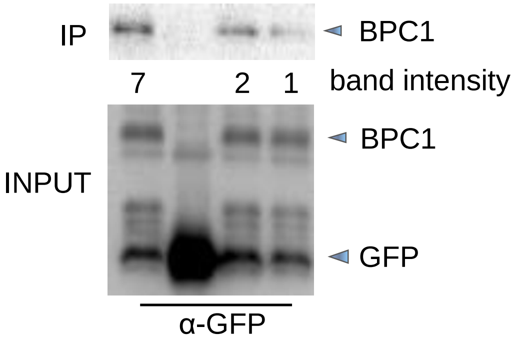

Supplementary Figure S3. DNA-pull down assay. *MtABI4* oligo and the mutated *MtABI4* oligo were labeled with biotin and incubated with the MtBPC1 protein. The interaction was measured by band intensity of the Western blot. GFP protein incubated with *MtABI4* oligo and BPC1 protein incubated with none-labeling oligo served as negative controls.

A

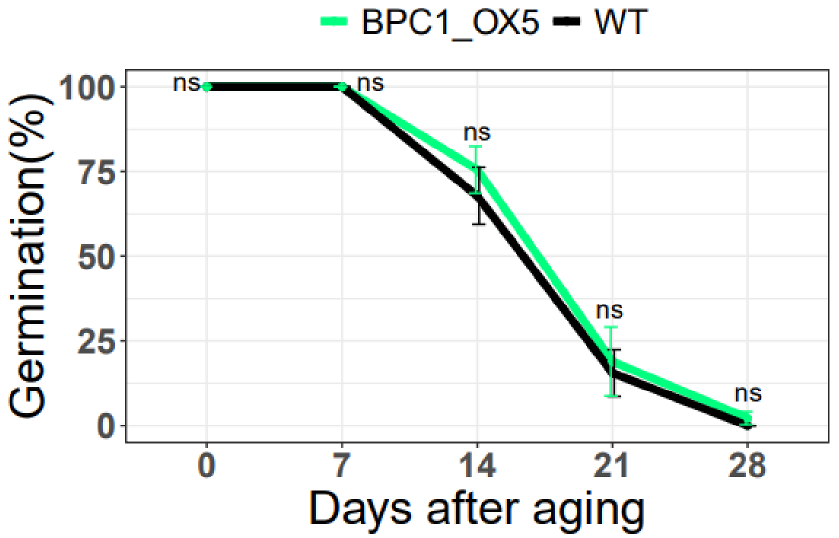

B

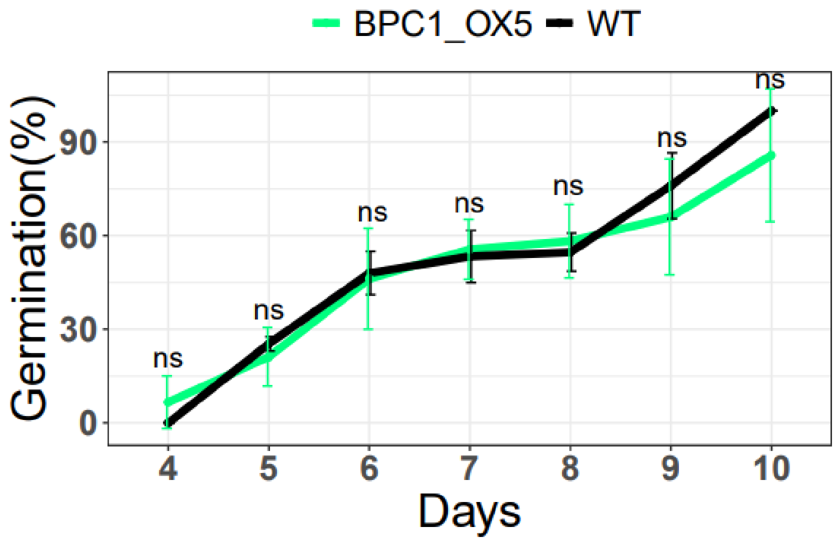

ABA treatment: 10 µM

Supplementary Figure S4. Evaluation of seed phenotypes of the BPC1_OX5 and WT mature seeds. (**A**). Survival curve. Aging was carred out at 75% RH and 35°C. Seed viability was tested by final germination (**B**). Germination curve in the presence of 10 µM ABA. ns. not significant, *P>0.05,* student t.test

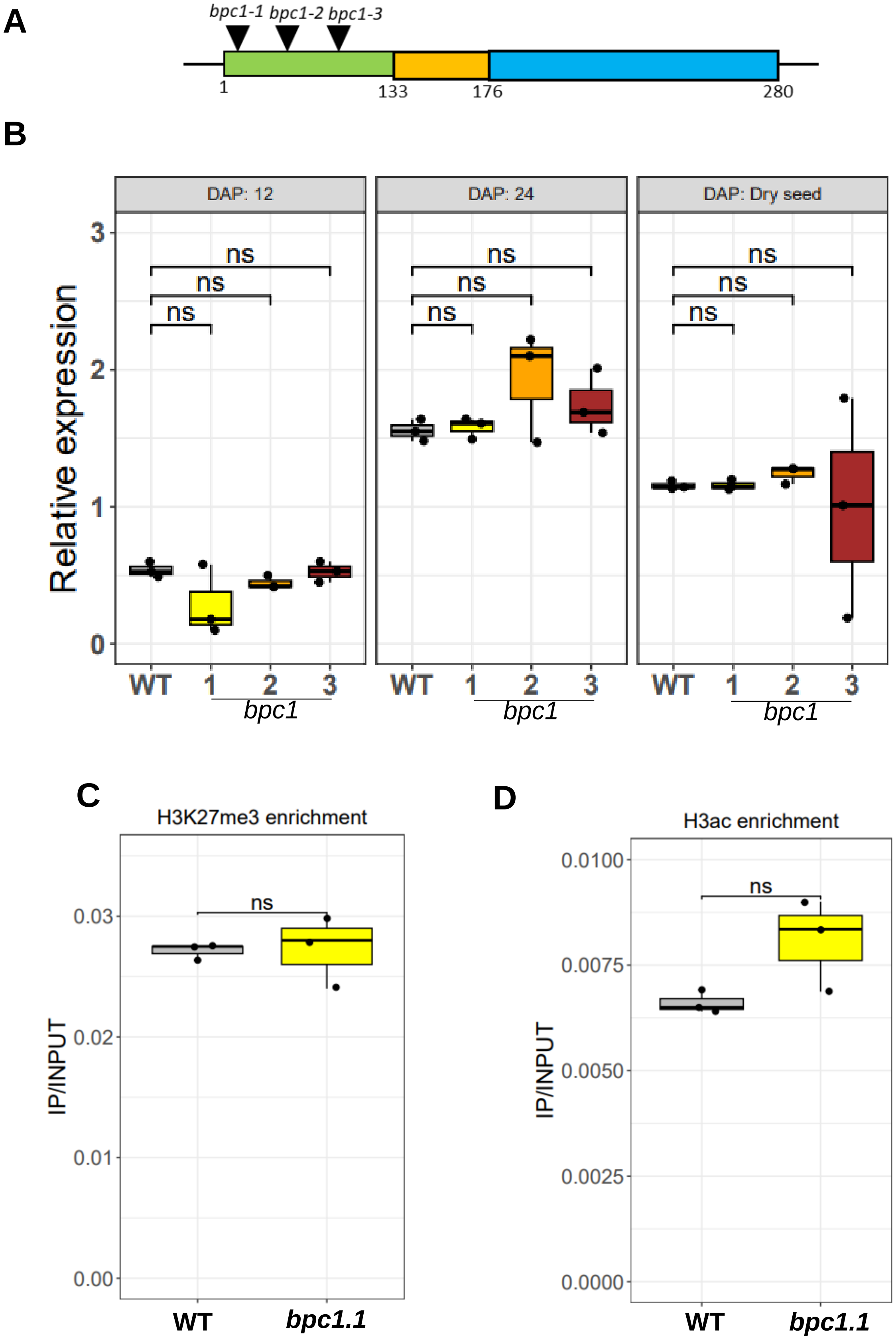

Supplementary Figure S5. Transcript level and histone mark modification of *MtABI4* in *bpc1* Tnt insertion mutants. **A**. Insertion position of the three Tnt mutants in the coding region of *MtBPC1*. **B**. Relative expression of *ABI4* in *bpc1* Tnt mutants at different stages of seed development. Ns. Not significant, *P>0.05,* student t.test. (**C**). H3K27me3 and (**D**) H3ac enrichment in the promotor of *ABI4* in *bpc1* line 1. DAP: days after pollination.

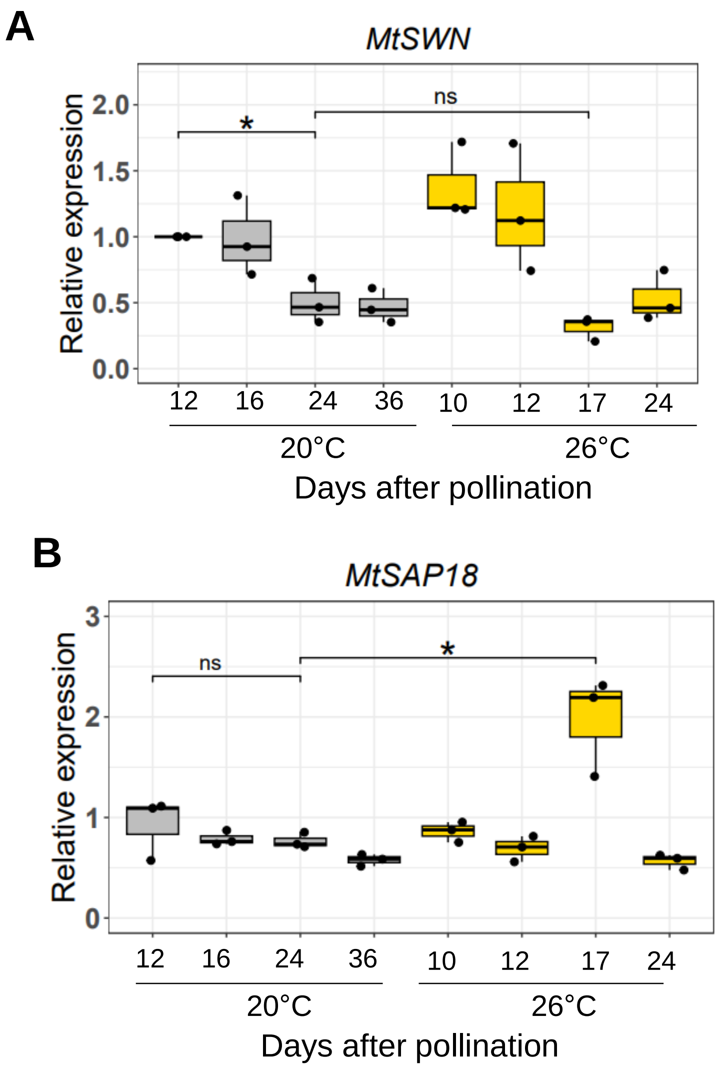

Supplementary Fig S6. Relative expression of *MtSWN* (A) and *MtSAP18* (B) at 20°C and 26°C by RT-qPCR. *. *P<0.05*, ns. Not significant, Ns. Not significant, *P>0.05,* student t.test

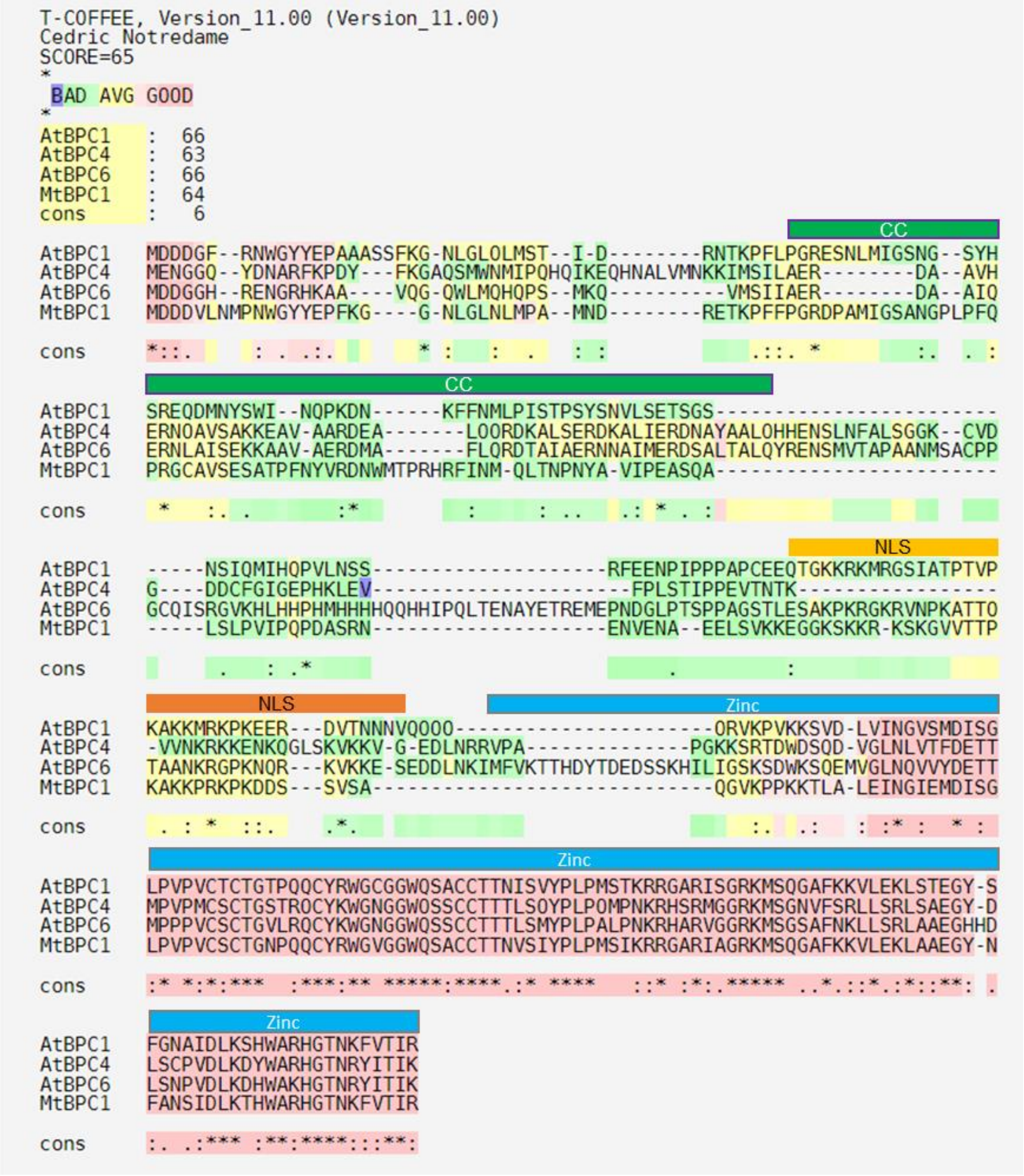

Supplementary Fig S7. Protein sequence alignment of AtBPC1, AtBPC4, AtBPC6, and MtBPC1

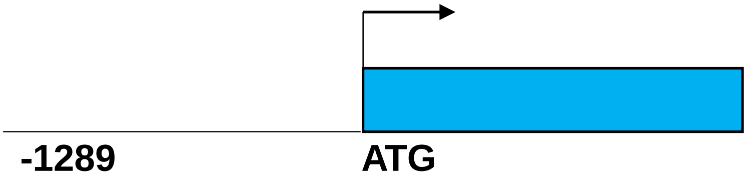

>MtrunA17Chr5 MtrunA17Chr5:36985575..36987332

AAGCGTTATAAATGCACACAACCGAATTGAAGACGATTAACAAAACGTCCATGTAATCGAATGAAGGTCGGACGA
TGAGACCAATCAAATACAAGTTGGGTGAGTATTTCTAATTAGGTCTAGATCTCACAAAGGGTGTTGGACTTAGAAT
TTCAAAAACAAGTAGAATTAACAATTCAAACTTAAAGACTTGTGTTTCATATATATATATATATATATATATATAGA
AGAGAGATTCGAATCAAAAAATGAGAATGCTGCAAAACATGAAGCACCACGTACAAACATGCACATTACTTGTAA
AAAAGCTTAATTACGAGAAATCGTATTAGATTTTATAGGACATGATCATTGGCCAAGGACACAAGTTTTGTTTATG
GAATAATTATTGTTCGTGTCATTTTCGACACAAATCAATCAAACTTATTATTTTATCGTAATTTGTCTTTTATCTTTAT
TTATAATTTATTAATGTTTCATTAGTTTAGTAAATGCAAAATTGTAACTAACATTCTTTTGAGTAGCTTAGGAAGTCA
AATCGAGGGACATAATAATTTCTTCTTTGACATAACCGTTTTGAAGTAAAAAAAAATAAAAAAAAATCGCATGTTGT
AGTTAACCTATTAGCTGAATCACCGACCAATTTTAAAGTTCGATAAAAACAATAGAAAATTAAAAATAAAAAGTTA
TGTTGTATTTGATGTTCAAATAAGATAAGTCAATAAATTATAGGTTAGTGTAATGGAGTATTAGTCTAGCATTCGTG
TAGTATAGTTTTTATAACTGTTTCGGAGCACAACATCACAATCCATCTCTTTCTAGAATACATTTGACTCAGAAACTG
TCACCTTATACAGAATCCCCGAGTTCCGCAATAACCCAATGCACTCATTGAGCGACGGTCACGAAATCCAACCATT
CAAGATCATCTTTCGTAACGGTCCATTTTACTCCACAATGGCTCCTTTCAATAGCCGTATAAATATCTCCCATCATCT
CATCACAAAGTACATGTTCTTTTCTCCTAAAAATCGCTCTCTATTTACTTTCTTGTTAACTTGAACTCAAAATGCTCAC
ATCGCTTCATCTAAAAGTCAAATTAAAAAGAACGTTATTCTACTTCGGCCTACTTTGAAGTCTATTAAGATCAATGA
AAACATGTTCAACCTTAGGTGGTAGCTAGCACTAATGTCATGCATTAATTAGTTACCTAGGAATTAGTTGTAATCGC
GGAATTAACTTTATCCCAAATAACACACCATCCTCGAAAGAGAAAACCTGACTATCAAGGTCAAGAAATAAAAAAT
ACAAAATTTAAATGATTCCAAATTCCCAAACCAATGTGCAACTATCTAATGGTGGTATCTCTATCTCTCTCTCTCTTT
TTTATTCACTTACAAAGCAAACAATCCCTTTCATTCATTACTCTCTCTCATATATAAAAAAATAAACAAAATTGCACA
CGCCCTATTCCACCATTAACATGAAACCACTTTCATTTCATCACAGAAGAAACAATAACACTGAACGAGGTAACCA
GTAGTCACTCTCCATCTTTTCTTTTTTCATTCATCTCTCACACACTCTTTCCTTTTAAA**ATG**

Supplementary Fig S8. The promoter sequence of *MtABI4* used as a bait construct for Yeast One Hybrid (Y1H) library screening. The 60-bp sequence used for Y1H validation is highlighted in yellow. The start codon is indicated in red.

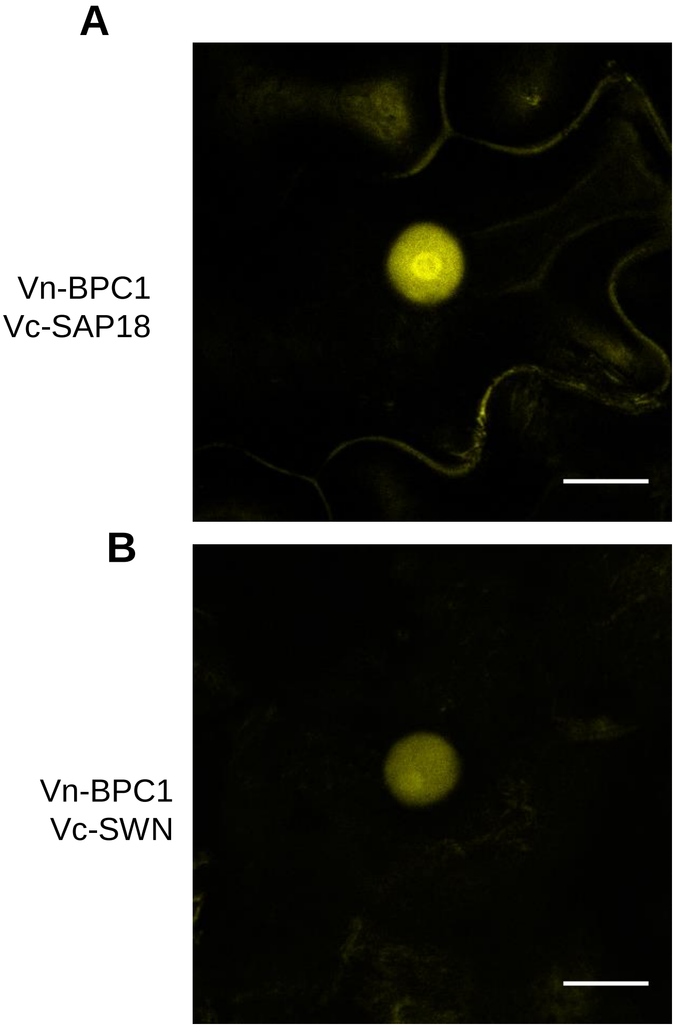

Supplementary Fig S9. Bi-FC of the interaction between BPC1-SWN **(A**) and BPC1-SAP18 (**B). Scale bar = 10 uM**
